# The Role of Frontostriatal Systems in Instructed Reinforcement Learning: Evidence From Genetic and Experimentally-Induced Variation

**DOI:** 10.3389/fnhum.2018.00472

**Published:** 2018-12-17

**Authors:** Nathan Tardiff, Kathryn N. Graves, Sharon L. Thompson-Schill

**Affiliations:** Department of Psychology, University of Pennsylvania Philadelphia, PA, United States

**Keywords:** reinforcement learning, prefrontal cortex, striatum, tDCS, dopamine, COMT, DAT

## Abstract

Instructions have a powerful effect on learning and decision-making, biasing choice even in the face of disconfirming feedback. Detrimental biasing effects have been reported in a number of studies in which instruction was given prior to trial-and-error learning. Previous work has attributed individual differences in instructional bias to variations in prefrontal and striatal dopaminergic genes, suggesting a role for prefrontally-mediated cognitive control processes in biasing learning. The current study replicates and extends these findings. Human subjects performed a probabilistic reinforcement learning task after receiving inaccurate instructions about the quality of one of the options. In order to establish a causal relationship between prefrontal cortical mechanisms and instructional bias, we applied transcranial direct current stimulation over dorsolateral prefrontal cortex (anodal, cathodal, or sham) while subjects performed the task. We additionally genotyped subjects for the COMT Val158Met genetic polymorphism, which influences the breakdown of prefrontal dopamine, and for the DAT1/SLC6A3 variable number tandem repeat, which affects expression of striatal dopamine transporter. We replicated the finding that the COMT Met allele is associated with increased instructional bias and further demonstrated that variation in DAT1 has similar effects to variation in COMT, with 9-repeat carriers demonstrating increased bias relative to 10-repeat homozygotes. Consistent with increased top-down regulation of reinforcement learning, anodal subjects demonstrated greater bias relative to sham, though this effect was present only early in training. In contrast, there was no effect of cathodal stimulation. Finally, we fit computational models to subjects' data to better characterize the mechanisms underlying instruction bias. A novel choice bias model, in which instructions influence decision-making rather than learning, was found to best account for subjects' behavior. Overall, these data provide further evidence for the role of frontostriatal interactions in biasing instructed reinforcement learning, which adds to the growing literature documenting both costs and benefits of cognitive control.

## Introduction

Successful learning and decision-making require a balance between exploiting prior information and learning from new experiences that may contradict it. One pervasive source of prior information in humans is instruction from others. Such instruction has clear benefits on both ontogenetic and historical timescales, allowing children to rapidly learn about the world and allowing culture and technology to develop and evolve (Tomasello, 1999). On an individual level, advice and information received from friends, professionals, and the media shape our view of the world and our choices.

The alternative to learning from advice and instruction is learning from direct experience of the world. One well-characterized method of learning from experience is reinforcement learning (RL), in which actions are selected so as to maximize reward (see Niv, 2009; Dolan and Dayan, 2013 for reviews). Recent work exploring the effects of instruction on RL has found that accurate advice can significantly improve performance (Biele et al., 2009; Doll et al., 2011). Yet such instruction is often detrimental when it is inaccurate. A potential consequence of inaccurate instruction and, more generally, inaccurate prior information, is *confirmation bias*, whereby data that are consistent with a prior hypothesis are sought, attended to, or valued over disconfirming data, which are neglected, filtered, or devalued (Nickerson, 1998). Confirmation bias is thought to be pervasive in human reasoning, affecting children and adults' scientific reasoning as well as that of professional scientists (Mahoney, 1977; Kuhn, 1989; MacCoun, 1998; Hergovich et al., 2010).

Biases have been induced in both social and non-social RL tasks utilizing various methods of information delivery. Information indicative of the moral character of computerized partners in a repeated trust game biases share decisions to “good” and “bad” partners despite identical behavior by the computer (Delgado et al., 2005; Fareri et al., 2012). Poor advice provided by fellow subjects impairs performance on the Iowa Gambling Task (Biele et al., 2009, 2011). Finally, in an RL task in which subjects learn to discriminate among pairs of probabilistically rewarded symbols, subjects instructed that a particular symbol is desirable persist in choosing that symbol more than would be expected given negative feedback, selecting it more frequently than symbols rewarded at an equal rate (Doll et al., 2009, 2011, 2014; Staudinger and Büchel, 2013). In sum, instructional biases appear to be persistent, and they are only partially ameliorated by feedback.

The neural substrates of instructed learning are still emerging, though as in uninstructed RL, frontostriatal areas are commonly implicated (Doll et al., 2009; Wolfensteller and Ruge, 2012). Neuroimaging has supported a role for prefrontal cortex (PFC) in representing instructions or prior information (Li et al., 2011; Fouragnan et al., 2013), with activity in instructed conditions found in dorsolateral PFC (DLPFC) and medial PFC. Connectivity analyses further support a role for PFC, reporting increased functional connectivity between frontal and striatal regions during instructed/prior knowledge conditions, consistent with top-down influence on striatal reward prediction errors (Li et al., 2011; Fouragnan et al., 2013).

Evidence of PFC altering striatal learning comports well with accounts of PFC-mediated cognitive control biasing or filtering information in other brain regions. Such top-down modulation focuses information processing on task-relevant information while suppressing irrelevant information (Shimamura, 2000; Miller and Cohen, 2001; Chrysikou et al., 2014). Performance should be optimal when the level of filtering is suitable to the demands of the task (Chrysikou et al., 2014). Consequently, increased top-down control can incur both costs and benefits. This is the case in instructed RL, where instruction-induced bias has been shown to vary according to individual differences in PFC dopaminergic tone caused by the catechol-*O*-methyltransferase (COMT) Val158Met genetic polymorphism (Doll et al., 2011). In particular, the Met allele, which has been shown to confer benefits in tests of working memory and cognitive control as compared to the Val allele (Durstewitz and Seamans, 2008; Witte and Flöel, 2012), is associated with a cost in the form of increased adherence to inaccurate instructions.

The goal of the present study was three-fold. First, we sought to replicate the effect of COMT on instructed reinforcement learning, providing further evidence for the role of PFC-mediated top-down control in biasing RL.

Second, we aimed to expand the understanding of the impact of striatal dopaminergic genes on instructed RL. While Doll et al. (2011) examined the effects of genetic polymorphisms specific to approach or avoidance learning in the striatum, we examined the effect of the DAT1/SLC6A3 variable number tandem repeat (VNTR), which affects striatal dopamine (DA) reuptake by altering expression of the dopamine transporter (DAT; Vandenbergh et al., 1992; Faraone et al., 2014). Though there are conflicting reports on the exact effects of the DAT1/SLC6A3 VNTR, a recent meta-analysis suggests that in healthy individuals the 9-repeat allele is associated with increased DAT expression in human striatum, and thus potentially more efficient reuptake of DA as compared to the 10-repeat variant (Faraone et al., 2014; cf. Costa et al., 2011). Striatal DA levels have previously been linked to cognitive flexibility (Cools and D'Esposito, 2009; Beeler et al., 2010; Garcia-Garcia et al., 2010), making DAT1 a plausible modulator of instructed RL.

Finally, while genetic and neuroimaging evidence is compelling, it falls short of establishing a causal role for PFC in biasing RL. We therefore hoped to establish this causal link by directly modulating PFC via transcranial direct current stimulation (tDCS). In keeping with a costs/benefits framework, we predicted that anodal stimulation—which has been successfully applied to PFC in order to improve cognitive control (Fregni et al., 2005; Cattaneo et al., 2011; Zaehle et al., 2011; Nozari and Thompson-Schill, 2013; Karuza et al., 2016)—would lead to increased bias due to increased top-down regulation. Cathodal stimulation over PFC has produced inconsistent results in cognitive domains (Jacobson et al., 2012; Nozari et al., 2014). However, supporting the costs/benefits framework, it has been linked to decreased working memory (Zaehle et al., 2011) and selective attention (Nozari et al., 2014; Zmigrod et al., 2016), but improved dual task performance (Filmer et al., 2013) and cognitive flexibility (Chrysikou et al., 2013). Therefore we tentatively predicted that cathodal stimulation would lead to decreased bias due to decreased top-down control of RL.

## Methods

### Subjects

One-hundred and twenty-six right-handed subjects (42 per condition, 80 female, M_age_ = 22.20 years) participated in the study, receiving $20 in compensation, regardless of performance. Informed consent was obtained from each subject in accordance with the University of Pennsylvania IRB. Subjects were randomly assigned to stimulation condition. We excluded a total of 23 subjects from the analyses for failure to meet the performance criteria described in section Performance Criteria (9 anodal, 6 cathodal, 8 sham), for a final sample of 103 (65 female, M_age_ = 21.84 years). Of these subjects, genotyping failed for one subject. For the Val158Met single-nucleotide polymorphism (SNP) of the COMT gene (rs4680), frequencies per allele in the final sample were 34:53:15 (Val/Val:Val/Met:Met/Met). For the DAT1/SLC6A3 VNTR in the 3′ untranslated region, frequencies per allele were 65:26:6:2:1:1:1 (10/10:9/10:9/9:10/11:8/9:8/8:6/10). Subjects were placed in a 10/10 group if they had two repeats of 10+; otherwise they were placed in a 9-repeat carrier group (67 10/10, 35 9c). Neither gene differed from Hardy-Weinberg equilibrium either across the whole sample (all *p*s > 0.14) or within racial/ethnic subgroups (all *p*s > 0.15; see Supplementary Tables 3–6 for sample demographic breakdown). There was no association between COMT and DAT genotypes (*p* > 0.35, Fisher's Exact Test), nor were there any associations between the two genes and stimulation condition (all *p*s > 0.3). For the dopamine genotype composite, the distribution of subjects was: 25:43:27:7 (0:1:2:3). The composite was also not significantly associated with stimulation condition (*p* = 0.09).

### Materials and Procedure

**Figure 1:**
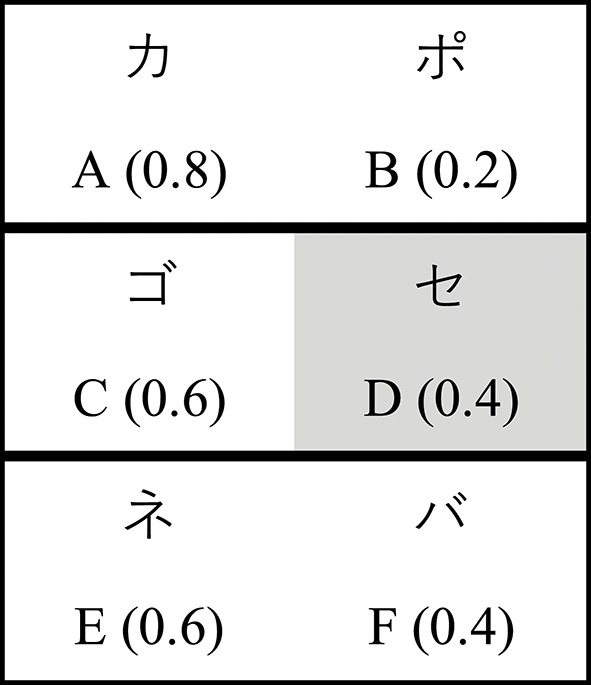
Stimuli (reward probabilities) for the instructed probabilistic selection task. Subjects are instructed that D is the best symbol.

Subjects completed an instructed probabilistic selection task (iPST), presented on a 13″ laptop computer via PsychoPy (Peirce, 2009). This task required subjects to learn the value of symbols initially presented in 3 pairs (AB, CD, EF; Figure 1). Within each pair, one symbol had a higher probability of reward. Symbols were rendered as Japanese Hiragana characters, and the assignment of Japanese character to underlying stimulus was randomized across subjects. During the instructions, each symbol was presented individually for 5 s to familiarize subjects with the stimuli. Crucially, when symbol D was presented the screen also displayed the following false advice: “This symbol has the best chance of being correct.”

During the training phase, subjects had to learn the value of each symbol via probabilistic feedback, which was delivered according to the symbol's underlying P(reward). Importantly, subjects were expected to learn to select the more highly rewarded symbol within each pair. Subjects completed 4 training blocks. Each block contained 20 repetitions of each pair, for a total of 60 trials per block and 240 total training trials. Trial order and feedback were randomized within each block. During the test phase, all possible symbol pairings were presented (e.g., AB, AC, AD, AE, AF, …) without feedback. Each pair was presented 6 times, for a total of 90 trials. Order was randomized across subjects. See section 1.1 of the Supplementary Material for further details regarding task design and presentation.

### Performance Criteria

Subjects had to meet the following performance criteria for the uninstructed symbols in order to be included in the analyses: ≥ 60% accuracy on the AB pair and ≥ 50% accuracy on the EF pair in at least one training block after the first block, with both criteria met in the same block. These criteria are similar to training phase learning criteria used in previous reports (Frank et al., 2007; Doll et al., 2009, 2011, 2014), but were relaxed slightly for AB to allow for additional variability in learning performance, given a previous report of tDCS effects on this pair (Turi et al., 2015). Subjects were also excluded if they failed to respond on >10% of training trials.

In addition to excluding subjects who failed to pay attention or learn, these criteria helped ensure that subjects with arbitrary biases for one of the uninstructed symbols were excluded from the analyses. However, to further protect against arbitrary affinities introducing bias into the between-group analyses, we further tested for the presence of genotype or stimulation differences in the first 10 training trials of the uninstructed training pairs. There were no significant effects (all *p*s > 0.10), indicating that none of our genotype or stimulation groups entered the training phase with arbitrary stimulus preferences.

### Genotyping

DNA samples were collected via Oragene saliva kits (DNA Genotek) and genotyped at the Penn Molecular Profiling Facility using standard procedures (see section 1.2 of the Supplementary Material).

### Transcranial Direct Current Stimulation

We delivered direct current via saline-soaked sponge electrodes with a 25 cm^2^ surface area. Current was generated by a continuous current stimulator (Magstim Eldith 1 Channel DC Stimulator Plus, Magstim Company Ltd., Whitland, Wales). In all conditions, 1.0 mA direct current was applied after a 30 s ramp-up period and was followed by a 30 s ramp-down. In the verum conditions, current was applied for 20 min. Stimulation was applied for only 30 s during sham. In the anodal condition, the anode was placed over F7, in accordance with the 10–20 international system, and the cathode was placed over the right supraorbital. This placement was reversed in the cathodal condition.

The F7-RSO montage was chosen because current modeling (HDExplore Software, v2.3, SOTERIX) suggested it would maximize current through DLPFC sites found to be active during instructed reinforcement learning conditions (Li et al., 2011; Fouragnan et al., 2013). Stimulation at F7 has been shown to modulate prefrontally-mediated cognitive control across a range of tasks (Lupyan et al., 2012; Chrysikou et al., 2013; Nozari et al., 2014). The procedure for each subject is outlined in Table 1. Stimulation began 180 s prior to the start of the first trial while subjects were presented with a fixation cross. Stimulation has not been shown to produce after-effects at 1.0 mA unless applied for at least 3 min, and thus this period gives stimulation time to take full effect (Nitsche and Paulus, 2000). Additionally, though stimulation ended after the training phase, after-effects have been reported up to an hour after stimulation lasting 9–13 min, so it is possible tDCS could directly affect performance at test in addition to its indirect effect through modifying performance during training (Nitsche and Paulus, 2001; Nitsche et al., 2003).

**Table 1:** Stimulation procedure and duration for verum stimulation (sham was identical except stimulation only lasted for 30 s, at the onset of the fixation period).

**Phase**	**tDCS**	**Duration**
Instructions	No	Variable
Fixation	Yes	3 min
Training	Yes	17 min
Test Instructions	No	Variable
Test	No	6 min

### Data Analysis

Statistical analyses were conducted in R (R Core Team, 2018) using logistic mixed models implemented in the lme4 package (Bates et al., 2015). By modeling both fixed and random effects, these models controlled for the non-independence inherent in within-subjects data. All models included random intercepts for subjects and random slopes for within-subjects variables and their interactions (Schielzeth and Forstmeier, 2009; Barr et al., 2013). When making between group comparisons of factors with more than two levels without planned comparisons, the significance of main effects and interactions were computed using the car package (Fox and Weisberg, 2011). *Post-hoc* comparisons were computed using the lsmeans package (Lenth, 2016). Significance levels for *post-hoc* comparisons were corrected using the Bonferroni-Holm method (Holm, 1979). Permutation tests were conducted via Monte Carlo sampling (1.0e6−1 permutations) using the perm package (Fay and Shaw, 2010).

### Computational Modeling

Reinforcement learning models were fit to each subject's data in order to evaluate hypotheses regarding the mechanisms of instructional bias. Models were fit by maximizing the log likelihood of the data using MATLAB's fmincon (Mathworks, MA, USA). To avoid local minima, each model fit was repeated 25 times from different random starting points, using RMSEARCH. All models were fit to both training and test phase data. For the training phase, fits were optimized to account for subjects' trial-wise choices; for the test phase, they were optimized to result in learned Q-values after training that best account for choices during test (Frank et al., 2007).

#### Standard Model

This model implements a standard Q-learning model with separate learning rates for gains and losses (Frank et al., 2007). The value of each stimulus is updated according to the following learning rule:

$$\begin{array}{l} Q_{t+1}\left(s\right)=Q_{t}\left(s\right)+{\left[\alpha_{\text{g}}*\delta_{t}\right]}_{+}+{\left[\alpha_{\text{l}}*\delta_{t}\right]}_{-}\\ \;\delta_{t}={r_{t}-Q}_{t}\left(s\right) \end{array}$$

where *Q*_*t*_(*s*) is the action value of stimulus *s* at trial *t*, *r*_*t*_ is the reward (0 for losses, 1 for gains), and δ_*t*_ is the reward prediction error. The learning rate α_g_ applies only to gain trials, while the learning rate α_l_ applies only to loss trials.

Choice in the standard model and subsequently described models was implemented via a softmax function:

$$\begin{array}{l} P_{t}\left(s_{1}\right)=\frac{\exp\left(\frac{Q_{t}\left(s_{1}\right)}{\beta }\right)}{\exp\left(\frac{Q_{t}\left(s_{1}\right)}{\beta }\right)\;+\;\exp\left(\frac{Q_{t}\left(s_{2}\right)}{\beta }\right)} \end{array}$$

where *P*_*t*_(*s*_1_) is the probability of choosing symbol *s*_1_ over symbol *s*_2_, and β is a temperature parameter determining the extent to which choice is deterministic vs. random.

For this model and subsequent models, we placed the following bounds on the parameters: α∈[0.002, 1]; β∈[0.06, 20]. The temperature parameter was additionally constrained by an empirical prior (Gershman, 2016): $\frac{1}{\beta }\sim \text{Gamma}\left(5.09,\;0.83\right)$. Q-values for all stimuli were initialized at 0.5.

#### Learning Bias Model (Doll et al., 2011)

The learning bias model is identical to the standard model in all respects except that when symbol D is chosen, the baseline learning rate is distorted as follows:

$$\begin{array}{l} Q_{t+1}\left(D\right)=Q_{t}\left(D\right)+{\left[\alpha_{\text{g}}*\alpha_{\text{bg}}*\delta_{t}\right]}_{+}+{\left[\frac{\alpha_{\text{l}}}{\alpha_{\text{bl}}}*\delta_{t}\right]}_{-} \end{array}$$

where α_bg_ increases the learning rate for instruction-consistent feedback (gains), α_bl_ diminishes the learning rate for instruction-inconsistent feedback (losses), and α_b·_ ∈ [1, 10]^1^.

#### Bayesian Hypothesis Testing Model (Doll et al., 2011)

This model accounts for the possibility that the bias lies not in learning the value of the instructed stimulus D but in the decision to choose D. In this case, the choice bias requires that learners achieve a certain level of confidence that D is rewarded below chance before they abandon it. This model implements a Bayesian Q-learner with *Q*_*t*_(*s*) ~ Beta[α_*t*_(*s*), *β*_*t*_(*s*)]. After reward feedback, posterior Q-value distributions are updated as:

$$\begin{array}{l} Q_{t+1}\left(s\right)\;\sim \text{ Beta}\left[\alpha_{t}\left(s\right)+r_{t},\;\beta_{t}\left(s\right)+\left(1-r_{t}\right)\right] \end{array}$$

which increments *α* by 1 after gains and *β* by 1 after losses. Additionally, after every trial the *α* and *β* counts decay toward uniform, controlled by free parameters *γ*_α_ and *γ*_*β*_; *γ*_·_ ∈ [0, 1] (0 is full decay and 1 is no decay). Choice is implemented by submitting the mean of each symbol's beta distribution to the softmax function above. Crucially, when the instructed stimulus is encountered, a decision bias is implemented as follows:

$$\begin{array}{l} P_{t}\left(s_{\text{alt}}\right)=\frac{\exp\left(\frac{0.5}{\beta }\right)}{\exp\left(\frac{0.5}{\beta }\right)\;+\;\exp\left(\frac{\mu_{t}\left(\text{D}\right)\;+\;\phi \;*\;\sigma_{t}\left(\text{D}\right)}{\beta }\right)} \end{array}$$

with *ϕ* ∈ [0, 20] and *P*_*t*_(D) = 1 − *P*_*t*_(*s*_alt_). This decision rule dictates that the mean value of D must be > ϕ standard deviations of D below chance before it is more probable that the alternative symbol, *s*_alt_, is chosen. Thus, the more certain the learner is of the value of D, the lower the bias.

#### Decision Bias Model

Though the Bayesian hypothesis testing model has provided a reasonable fit to some subjects' training data and has been shown to be sensitive to individual differences, it has not overall outperformed the standard model in explaining training phase performance (Doll et al., 2009, 2011). It also compares the value of D to chance instead of to the value of the alternative stimulus, making it less effective as a possible model of test phase performance. Furthermore, interpretation of this model in comparison to the standard uninstructed model is complicated by the fact that they are not nested models. Therefore, we also implemented a novel decision bias model. This model uses the standard Q-learner described above, but the softmax decision rule is modified for choices involving the instructed stimulus in a manner similar to the Bayesian hypothesis testing model:

$$\begin{array}{l} P_{t}\left(s_{\text{alt}}\right)=\frac{\exp\left(\frac{Q_{t}\left(s_{\text{alt}}\right)}{\beta }\right)}{\exp\left(\frac{Q_{t}\left(s_{\text{alt}}\right)}{\beta }\right)\;+\;\exp\left(\frac{Q_{t}\left(\text{D}\right)\;+\;\rho }{\beta }\right)} \end{array}$$

with ρ∈[0, 1]. The free parameter ρ determines how much greater the value of the alternative symbol must be before it is more probable that it is chosen over D. Therefore, unlike the Bayesian model, this model: (a) assumes a fixed bias; (b) compares the value of D to the alternative symbol, making it more appropriate as a model of test phase choice; and (c) contains the standard model as a special case (ρ = 0), ensuring differences in fit will be attributable to the presence of the bias and not to differences in the learner.

#### Model Comparison

Goodness of fit was assessed using Akaike information criteria (AIC). We additionally submitted the AIC values to a Bayesian random effects analysis, which assumes there is a distribution of models in the population and attempts to identify which model is most prevalent. The quantity resulting from this analysis is a protected exceedance probability (PEP), which is the probability that a given model is the most frequent in the population, above and beyond chance (Rigoux et al., 2014). PEPs were computed using the VBA toolbox (Daunizeau et al., 2014). Model comparison was then made on the basis of both AIC and PEPs.

## Results

We begin by reviewing general performance across the sample. We then examine genotypic differences in instructional bias. To this end, we first attempt to replicate the effect of COMT genotype. We then extend the investigation of the influence of dopaminergic genes on instructional bias to the DAT1 gene. In brief, we partially replicated the effect of COMT and found effects of DAT1 on instructional bias as well. Motivated by these findings, we next ask whether a dopamine composite variable constructed from the COMT and DAT variables captures additional aspects of performance. These analyses demonstrated an overall graded effect of the dopamine composite on performance and also uncovered a small group of subjects who demonstrated more extreme bias. We then ask if we can causally manipulate instructional bias with tDCS, finding that anodal stimulation had a small but significant effect on performance during training. Finally, we fit computational models to test potential mechanisms underlying instructional bias, finding evidence in favor of a model incorporating a bias on the decision to choose the instructed stimulus, rather than a bias on the learned value of the instructed stimulus.

### General Performance: Training Phase

#### Instructed Learning

We first conducted analyses of choice behavior during training. In all analyses, accuracy was binary coded (incorrect: 0, correct: 1), where correct is defined as choosing the stimulus with the higher probability of reward, regardless of whether it was rewarded on that trial. Trial Type was treatment coded (CD: 0, EF: 1). This coding allows direct assessment of how much instruction biased learning. Block was reverse Helmert coded in order to capture learning-related changes in the mean level of responding across training (i.e., Block 2 was compared to Block 1, Block 3 was compared to the mean of Blocks 1 and 2, and Block 4 was compared to the mean of all prior blocks). We assessed the effects of genotype and stimulation both by examining performance on the CD trials alone, and by contrasting performance on CD with the equally rewarded but uninstructed EF pair. Given our between-subjects design, this latter contrast serves to account for additional variance in learning unrelated to instructional control. Therefore, instructed training models included all two-way and three-way interactions of genotype or tDCS condition, Trial Type, and Block.

Subjects were below chance on the CD pair (β = −0.27, *z* = −2.86, *p* = 0.004). Performance was significantly better on the EF pair (β = 0.93, *z* = 6.96, *p* < 0.0001), validating the success of the instructional manipulation. Despite poor overall performance on the CD pair, subjects continued to learn away from the instructions throughout training, as demonstrated by the significance of all three Block regressors (Block 2 vs. 1: β = 0.32, *z* = 2.81, *p* = 0.005; Block 3 vs. (1, 2): β = 0.24, *z* = 2.34, *p* = 0.02; Block 4 vs. (1,2,3): β = 0.31, *z* = 3.13, *p* = 0.002).

#### Uninstructed Learning

Variable coding in uninstructed training models was the same as above, except Trial Type was effect coded (AB: 1, EF: −1). The three-way interactions were not included in these models as there were no hypotheses relevant to these contrasts.

Subjects performed significantly above chance on uninstructed trials (β = 1.12, *z* = 14.67, *p* < 0.0001). There was an effect of Trial Type (β = 0.46, *z* = 10.55, *p* < 0.0001), indicating that subjects performed significantly better on the AB pair over the EF pair, in line with the relative difficulty of the two discriminations. Subjects continued to learn throughout training, though the magnitude of this effect was numerically smaller in later blocks (Block 2 vs. 1: β = 0.40, *z* = 4.79, *p* < 0.0001; Block 3 vs. (1, 2): β = 0.22, *z* = 2.74, *p* = 0.006; Block 4 vs. (1, 2, 3): β = 0.18, *z* = 2.17, *p* = 0.03). There was additionally a Trial Type x Block 2 vs. 1 interaction (β = 0.31, *z* = 3.97, *p* < 0.0001), indicating a steeper learning trajectory for AB over EF during the initial blocks of the task, which again is unsurprising given the relative ease of the AB discrimination.

### General Performance: Test Phase

The training and test phases are purported to represent different processes subserved by different neural systems (Frank et al., 2007). While the training phase is supposed to reflect hippocampally- and frontally-mediated memory and hypothesis-testing processes, the test phase is designed to give a “purer” measure of striatally-learned reinforcement values. The standard approach to assessing performance at test is to examine performance on trials in which a stimulus of interest is included in novel pairings, giving an estimate of how well underlying reward values were learned during training.

Two measures from the literature were used to assess the effect of instruction on test phase performance (Doll et al., 2014). The first analysis compared performance on Avoid-D (AD, DE) vs. performance on Avoid-F (AF, CF). For both measures, the target stimulus should not be chosen, as it has been paired with stimuli that had a higher probability of reward during training. Given that D and F had identical reward probabilities during training, subjects should perform equally well on both measures. However, if instruction biased the ultimate reward values subjects learned, or if subjects' choices continue to be biased at test, they should avoid D at a lower rate than they avoid F.

Choice Type was entered as an effect-coded factor (Avoid-D: 1, Avoid-F: −1) in a logistic mixed effects model of choice performance. The intercept was significant (β = 0.73, *z* = 5.53, *p* < 0.0001), indicating that subjects' overall avoidance of D and F was above chance. There was also a main effect of Choice Type (β = −0.61, *z* = −5.43, *p* < 0.0001). As expected, subjects showed a confirmation bias effect, avoiding D significantly less than they avoided the equally rewarded symbol F.

The second analysis of instructed learning examined performance on DF trials in order to directly compare the relative subjective value of the two stimuli. A greater effect of instruction on learning, and thus a greater bias, should be associated with an increased tendency to choose D over F.

In this model, choice on DF trials was the dependent variable (D: 1, F: 0). The intercept was significant (β = 1.58, *z* = 5.86, *p* < 0.0001). Subjects demonstrated a strong bias—they were almost five times more likely to choose D, as indicated by an odds ratio (OR) of 4.86. In sum, our training and test results replicate previous investigations (Doll et al., 2009, 2011, 2014) and confirm that the instructional manipulation was successful.

### COMT: Training Phase

#### Instructed Learning

We next sought to replicate the effect of the COMT Met allele on adherence to the instructions during training (Doll et al., 2011). COMT genotype was effect coded. All other variables were coded as above.

**Figure 2:**
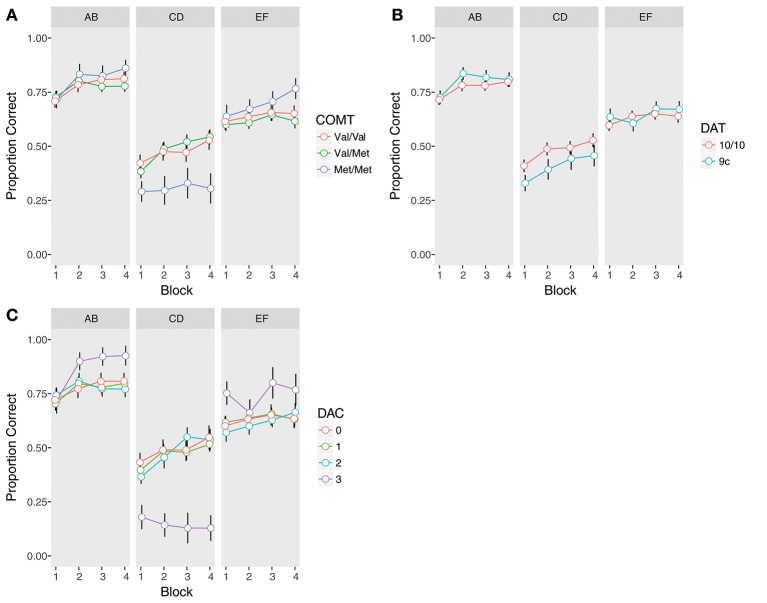
Training phase performance by trial type (AB, CD, EF) and genotype. Accuracy is defined as the proportion of time the symbol with the higher reward probability was chosen, regardless of whether it was rewarded or not. Error bars are standard errors of the mean. **(A)** COMT, **(B)** DAT, **(C)** Dopamine composite (DAC).

There was a significant COMT x Trial Type interaction (χ^2^_(2)_ = 13.94, *p* = 0.0009). Met homozygotes were significantly worse overall on the instructed pair (Figure 2A, Supplementary Table 7), as compared to both heterozygotes (β = −0.98, *z* = −3.70, *p*_*corrected*_ = 0.001) and Val homozygotes (β = −0.92, *z* = −3.26, *p*_*corrected*_ = 0.006). Met homozygotes also demonstrated better performance at a trend level on the uninstructed EF pair compared to Val/Met subjects, but this did not survive correction for multiple comparisons (β = 0.41, *z* = 1.87, *p* = 0.06, *p*_*corrected*_ = 0.25). Notably, no other comparisons reached significance, including the comparison of instructed performance between Val/Met and Val/Val subjects (all *p*s > 0.2), indicating impaired performance was specific to Met homozygotes.

Because our Met/Met group was somewhat small (*N* = 15) due to the low frequency of this genotype in the general population (Auton et al., 2015), we took a number of additional steps to ensure these results were not spurious. First, we reran our analyses comparing Val homozygotes to Met carriers (Metc), which was also the analysis performed by Doll et al. (2011). In this case, we failed to replicate the effect of Met-carrier status on instructed learning. The Metc x Trial Type interaction was not significant (χ^2^_(1)_ = 0.16, *p* = 0.69), nor were there any other significant effects of Met carrier status (all *p*s > 0.42). We then asked whether the full COMT model or the Metc model provided a better fit to the data, finding that the COMT model was a modestly better fit, despite including additional parameters (AIC_COMT_ = 19966, AIC_Metc_ = 19969). Finally, we conducted permutation tests on CD trials, averaged across all blocks, to further guard against the possibility that our Met homozygote results could have arisen under the null. Confirming our results, Met homozygotes' performance was reliably below the mean on CD trials (*p* = 0.004), and this group performed worse than both Val homozygotes (*p*_*corrected*_ = 0.006) and heterozygotes (*p*_*corrected*_ = 0.001). We therefore utilize the full breakdown of COMT genotype for the remainder of the results.

#### Uninstructed Learning

In contrast to instructed learning, we found no effects of COMT genotype on uninstructed learning (all *p*s > 0.2; Figure 2A, Supplementary Table 8).

### COMT: Test Phase

**Figure 3:**
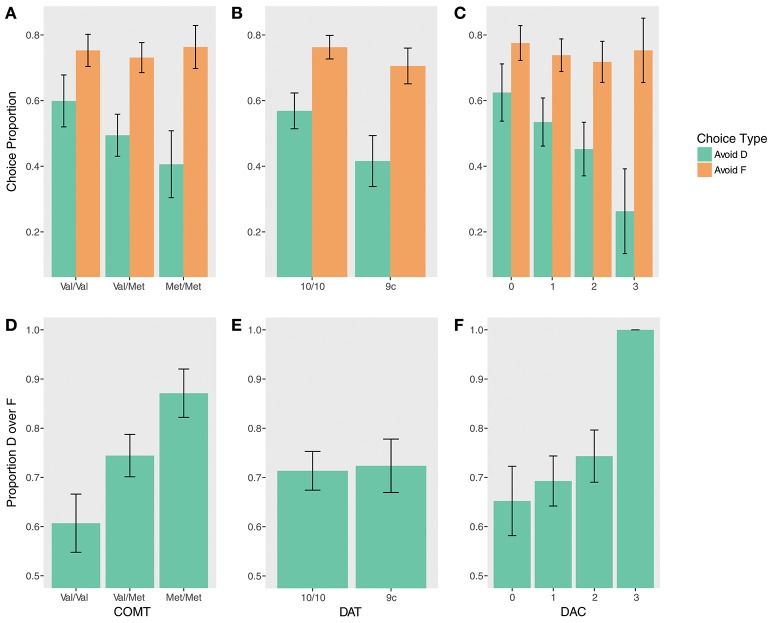
Test phase performance by genotype. (Top) Accuracy avoiding D (instructed) and F (uninstructed) when paired with stimuli at test that had a higher reward probability during training for **(A)** COMT, **(B)** DAT, **(C)** DAC. (Bottom) Proportion by which D was chosen over F at test for **(D)** COMT, **(E)** DAT, **(F)** DAC.

Instructed test phase performance demonstrated evidence of a gene-dose effect (Figures 3A,D). COMT status significantly predicted performance on DF trials (χ^2^_(2)_ = 9.06, *p* = 0.01). Val homozygotes were less likely to choose D on DF trials compared to heterozygotes (β = −1.11, *z* = −2.09, *p*_*corrected*_ = 0.07) and to Met homozygotes (β = −2.28, *z* = −2.82, *p*_*corrected*_ = 0.01). There was no significant difference between Val/Met and Met/Met groups, but Met/Met subjects were numerically more likely to choose D (β = 1.18, *z* = 1.53, *p*_*corrected*_ = 0.13). Supporting this pattern, an exploratory gene-dose analysis demonstrated a significant linear effect of the number of Met alleles on choosing D over F (β = 1.60, *z* = 3.01, *p* = 0.003).

There were no significant effects of COMT genotype on the Avoid-D/Avoid-F measure (all *p*s > 0.17), but quantitatively, differences were indicative of a similar gene-dose relationship on Avoid-D. An exploratory gene-dose analysis demonstrated a trend-level Met x Trial Type interaction (β = −0.44, *z* = −1.86, *p* = 0.06). While increasing Met alleles negatively predicted performance on Avoid-D (β = −0.90, *z* = −2.09, *p*_*corrected*_ = 0.07), there was no relationship with uninstructed Avoid-F (β = −0.02, *z* = −0.05, *p*_*corrected*_ = 0.96).

The above results refine, but only partially replicate, the effect of COMT genotype on instructed RL. While Doll et al. (2011) found that Met carriers demonstrate greater instructional bias relative to Val homozygotes during training, we found increased bias exclusively for Met homozygotes. Our COMT test phase results provide novel evidence for a gene-dose effect, though differences on the Avoid-instructed measure were not as robust as reported previously. The prior report included a somewhat greater percentage of Met homozygotes out of all Met carriers (28.3%) than the present study (22.1%), which could have impacted the results given that instructional bias appears to be strongest in the former group. Additionally, a number of methodological differences could have contributed to these discrepancies. These differences aside, as COMT is thought to be particularly and differentially important to the regulation of prefrontal dopamine levels (Durstewitz and Seamans, 2008; Tunbridge, 2010), the present findings further implicate prefrontal cortex in biasing responding to instructed stimuli at both training and test.

### DAT: Training Phase

Expanding the investigation of the effect of dopaminergic genes on instructional bias, we next examined the effect of DAT1 genotype. In our regression models, DAT was simple coded with 10/10 homozygotes as the reference (9c: 0.5, 10/10: −0.5).

As compared to 10-repeat homozygotes, 9-repeat carriers were significantly worse on the instructed pair (β = −0.43, *z* = −2.17, *p* = 0.03; Figure 2B, Supplementary Table 9). There was also a trend-level DAT x Trial Type interaction (β = 0.53, *z* = 1.91, *p* = 0.056). While 9-repeat carriers were worse on the CD pair, there was no difference between genotypes on the EF pair (*p* > 0.5). There were no interactions between DAT and Block, indicating similar learning trajectories in both groups (all *p*s > 0.4). Nor were there any effects of DAT on uninstructed learning (all *p*s > 0.4; Figure 2B, Supplementary Table 10).

### DAT: Test Phase

There was no effect of DAT on DF trials (*p* = 0.74; Figure 3E). There was a main effect of DAT on Avoid-D/Avoid-F (β = −0.66, *z* = −2.40, *p* = 0.02; Figure 3B) in the absence of a significant interaction (*p* > 0.17), suggesting that 9-repeat carriers were significantly worse overall on these measures. However, the effect seems to be driven primarily by worse performance on Avoid-D (Avoid-D: β = −0.49, *z* = −2.30, *p*_*corrected*_ = 0.04; Avoid-F: β = −0.17, *z* = −1.18, *p* = 0.24).

Remarkably, though DAT plays little role in cortical DA clearance (Sulzer et al., 2016), it appears to be equally if not more predictive of training than test phase performance, the former of which is putatively more reliant on prefrontal function (Frank et al., 2007). This result is surprising, given that investigations assessing other striatal genes have found that striatal genotypic effects in both instructed and uninstructed learning are confined to the test phase only (Frank et al., 2007; Doll et al., 2011). Previous work has indicated that there is a reciprocal relationship between prefrontal and striatal DA, with more prefrontal DA leading to more cognitive stability, while more striatal DA leads to more cognitive flexibility (Cools and D'Esposito, 2009). Motivated by this and by prior studies in which composites of multiple DA genes have shown better predictive power than single genes (Nikolova et al., 2011; Kohno et al., 2016), we next asked whether a composite DA variable would better predict instructional bias.

### DA Composite: Training Phase

To produce the DA composite (DAC), we recoded the COMT and DAT variables according to putative prefrontal-striatal DA balance (COMT: Val/Val = 0, Val/Met = 1, Met/Met = 2; DAT: 10/10 = 0, 9c = 1), and then summed the two variables. The resulting composite ranged between 0 (low frontal DA, high striatal DA) and 3 (high frontal DA, low striatal DA).

Reexamining training phase performance (Figure 2C, Supplementary Table 11), we found a significant effect of DAC (χ^2^_(3)_ = 11.02, *p* = 0.01), superseded by a significant DAC x Trial Type interaction (χ^2^_(3)_ = 29.56, *p* < 0.0001). *Post-hoc* comparisons revealed that the DAC3 group was significantly and uniquely impaired in learning away from the instructions compared to the other three groups (DAC3 vs. DAC 0: β = −2.03, *z* = −5.52, *p*_*corrected*_ < 0.0001; DAC3 vs. DAC 1: β = −1.96, *z* = −5.56, *p*_*corrected*_ < 0.0001; DAC3 vs. DAC 2: β = −1.99, *z* = −5.46, *p*_*corrected*_ < 0.0001). In contrast, DAC3 subjects demonstrated better performance on EF, though this did not survive correction for multiple comparisons: (DAC3 vs. DAC 0: β = 0.62, *z* = 1.94, *p* = 0.053, *p*_*corrected*_ = 0.42; DAC3 vs. DAC 1: β = 0.58, *z* = 1.92, *p* = 0.056, *p*_*corrected*_ = 0.42; DAC3 vs. DAC 2: β = 0.68, *z* = 2.14, *p* = 0.03, *p*_*corrected*_ = 0.29). No other comparisons between DAC groups were significant (all *p*s > 0.6). The DAC x Trial Type interaction was already present in the first block of training, suggesting it was not the result of extensive learning (χ^2^_(3)_ = 15.89, *p* = 0.001). Nor was it ameliorated by additional training, as the DAC3 group was the only group to show no evidence of learning away on CD from the first block to the last (DAC 0: β = 0.53, *z* = 2.00, *p*_*corrected*_ = 0.09; DAC 1: β = 0.57, *z* = 2.83, *p*_*corrected*_ = 0.01; DAC 2: β = 0.80, *z* = 3.11, *p*_*corrected*_ = 0.008; DAC 3: β = –0.59, *z* = −1.03, *p*_*corrected*_ = 0.30).

There were no significant differences between DAC groups in the analysis of uninstructed learning (all *p*s > 0.3), though as with EF, the DAC3 group's performance was quantitatively better on AB (Figure 2C, Supplementary Table 12). These differences in uninstructed learning are intriguing given that they are in the opposite direction of the instructed effect, but given the small sample size of the DAC3 group (*N* = 7) due to the lower prevalence of both the COMT Met allele (Auton et al., 2015) and the DAT 9-repeat variant (Vandenbergh et al., 1992; Doucette-Stamm et al., 1995) in the general population, this study may not have had the statistical power to determine whether such small effects are reliable.

As with the COMT Met/Met results, because of the small sample size of the DAC3 group, we again took efforts to ensure these results did not arise by chance. First, we repeated the analysis with a modified DA composite created by summing the Metc and DAT variables (Metc: Val/Val = 0, Met carrier = 1; DAT: 10/10 = 0, 9c = 1), producing three DACmetc groups *N*s = 25:51:26 (0:1:2). Repeating our analysis of instructed learning, we failed to find any effects of DACmetc (all *p*s > 0.21). However, the full DAC model provided a much better fit to the data, despite including additional parameters (AIC_DAC_ = 19958, AIC_DACmetc_ = 19974), and also provided a better fit than both the COMT and DAT instructed learning models (AIC_COMT_ = 19966, AIC_DAT_ = 19961). Permutation tests on the average performance on CD trials across training also support the results of the regression analysis. DAC3 subjects were reliably below the mean on CD trials (*p* < 0.0001), and this group performed worse than all other DAC groups (DAC3 vs. DAC0: *p*_*corrected*_ = 0.0001, DAC3 vs. DAC1: *p*_*corrected*_ < 0.0001, DAC3 vs. DAC2: *p*_*corrected*_ = 0.0001). Given that it is highly unlikely that seven randomly chosen subjects would have performance at the level of the DAC3 group, we utilize the full DAC composite for the remainder of the results.

### DA Composite: Test Phase

While there was only a marginal main effect of DAC on Avoid-D/Avoid-F (χ^2^_(3)_ = 6.62, *p* = 0.085), a gene-dose analysis revealed a significant linear effect of DAC (β = −0.86, *z* = −2.56, *p* = 0.01) qualified by a DAC x Choice Type interaction (β = −0.62, *z* = −2.16, *p* = 0.03). DAC status was negatively associated with avoiding D; it showed no relationship to avoiding F (Avoid-D: β = −1.47, *z* = −2.88, *p*_*corrected*_ = 0.008; Avoid-F: β = −0.24, *z* = −0.68, *p*_*corrected*_ = 0.50; Figure 3C). DF trials revealed a similar pattern; though there was no main effect of DAC (χ^2^_(3)_ = 1.12, *p* = 0.77), there was a significant gene-dose effect, with increasing choice of the instructed stimulus with increasing DAC status (β = 1.72, *z* = 2.53, *p* = 0.01). This effect appears to be driven primarily by the DAC3 group, all seven of whom remarkably chose D over F 100% of the time (Figure 3F).

In sum, there was graded effect of DAC on test phase performance, with increasing frontal (decreasing striatal) DA predicting greater adherence to the instructions. This graded relationship was punctuated by the performance of the DAC3 group, who, as during training, demonstrated substantially greater instructional bias.

Taken together, the genotyping results implicate prefrontal cortex, and in particular the balance between prefrontal and striatal dopamine, in modulating instructed RL. This pattern motivates asking our next question: Does experimentally manipulating prefrontal function via tDCS alter the magnitude of instructional bias?

### tDCS: Training Phase

#### Instructed Learning

To examine the main hypotheses of the study—that anodal stimulation will increase confirmation bias, while cathodal stimulation may decrease it—our focal analyses concerned the contrasts of Anodal vs. Sham stimulation and Cathodal vs. Sham stimulation. These contrasts include Condition, or the overall effect of stimulation compared to Sham on instructed choice, and Condition x Trial Type, which allows for the same assessment while controlling for performance on EF. For a more fine-grained investigation of the time course of learning, we additionally examined the Condition x Block interactions, which indicate whether stimulation altered the extent to which subjects learned away from the instructions across training blocks, and the Condition x Trial Type x Block interactions, which allow for the same assessment while controlling for performance on EF. Condition was simple coded with sham as the reference (Anodal: 2/3 −1/3, Cathodal: −1/3 2/3, Sham: −1/3 −1/3).

**Figure 4:**
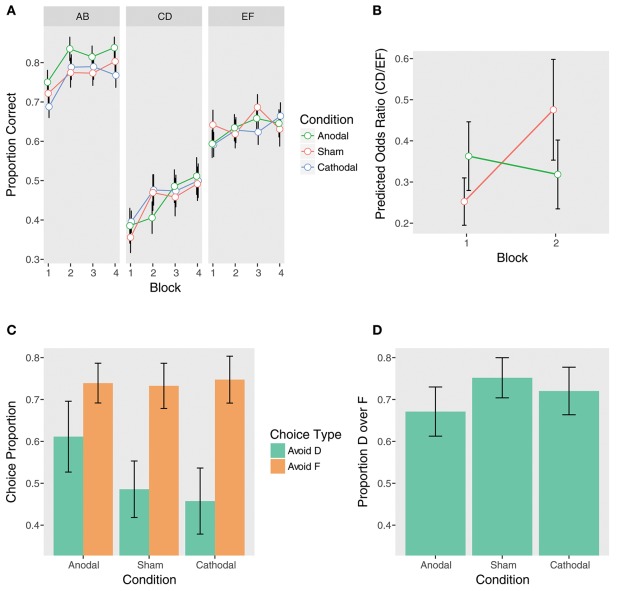
Performance at training (top) and test (bottom) by tDCS stimulation condition. **(A)** Training phase performance by trial type. **(B)** The effect of anodal stimulation on instructed reinforcement learning. Points are predicted odds ratios for the CD/EF contrast by block and condition. This contrast reflects performance on CD controlling for performance on EF, giving a purer measure of the effect of instructions on choice. Lines represent the two-way Trial Type x Block interactions within each condition. Error bars are standard errors of the parameter estimates. While the sham group demonstrated significant learning away from the instructions from Block 1 to Block 2, the anodal group did not, and this interaction was significant (see section tDCS: Training Phase: Instructed Learning). **(C)** Avoid-D/Avoid-F. **(D)** DF trials.

We first examined the contrasts between anodal and sham stimulation. Supporting our hypothesis, there was a significant Anodal vs. Sham x Trial Type x Block 2 vs. 1 interaction (β = 0.76, *z* = 2.22, *p* = 0.03). When controlling for performance on EF, the sham group demonstrated significant learning away from the instructions from Block 1 to Block 2 on CD, while the anodal group did not (Sham: β = 0.63, *z* = 2.60, *p*_*corrected*_ = 0.046; Anodal: β = −0.13, *z* = −0.53, *p*_*corrected*_ = 1.00). The sham group nearly doubled their performance (OR = 1.88), but the anodal group demonstrated essentially no learning (OR = 0.88; Figures 4A,B and Table 2). Examining performance on CD without adjusting for EF, the Anodal vs. Sham x Block 2 vs. 1 interaction was at trend (β = −0.45, *z* = −1.59, *p* = 0.11). As above, the sham group showed significant learning from Block 1 to Block 2, while the anodal group did not (Sham: β = 0.53, *z* = 2.68, *p*_*corrected*_ = 0.04; Anodal: β = 0.08, *z* = 0.42, *p*_*corrected*_ = 1.00). In contrast, neither group demonstrated significant learning from Block 1 to Block 2 on EF (Sham: β = −0.10, *z* = −0.64, *p*_*corrected*_ = 1.00; Anodal: β = 0.21, *z* = 1.33, *p*_*corrected*_ = 0.74).

**Table 2:** Mixed effects logistic regression model of the effect of instruction (CD vs. EF) and tDCS on training phase performance.

**Predictor**	**β**	***OR^a^***	***z***	***p***
**Intercept**	**−0.27**	**0.76**	**−2.86**	**0.004**
Anodal vs. Sham	−0.01	0.99	−0.04	0.97
Cathodal vs. Sham	0.09	1.10	0.40	0.69
**Trial Type**	**0.93**	**2.53**	**6.96**	**<0.0001**
**Block 2 vs. 1**	**0.32**	**1.38**	**2.81**	**0.005**
**Block 3 vs. (1, 2)**	**0.24**	**1.27**	**2.35**	**0.02**
**Block 4 vs. (1, 2, 3)**	**0.31**	**1.36**	**3.13**	**0.002**
Anodal vs. Sham x Trial Type	−0.05	0.95	−0.15	0.88
Cathodal vs. Sham x Trial Type	−0.17	0.84	−0.54	0.59
Anodal vs. Sham x Block 2 vs. 1	−0.45	0.64	−1.59	0.11
Anodal vs. Sham x Block 3 vs. (1, 2)	0.18	1.20	0.73	0.47
Anodal vs. Sham x Block 4 vs. (1, 2, 3)	0.08	1.07	0.31	0.75
Cathodal vs. Sham x Block 2 vs. 1	−0.19	0.83	−0.69	0.49
Cathodal vs. Sham x Block 3 vs. (1, 2)	−0.10	0.91	−0.41	0.68
Cathodal vs. Sham x Block 4 vs. (1, 2, 3)	−0.17	0.85	−0.70	0.48
Trial Type x Block 2 vs. 1	−0.22	0.80	−1.58	0.11
Trial Type x Block 3 vs. (1, 2)	−0.03	0.97	−0.22	0.82
Trial Type x Block 4 vs. (1, 2, 3)	−0.16	0.85	−1.11	0.27
**Anodal vs. Sham x Trial Type x Block 2 vs. 1**	**0.76**	**2.15**	**2.22**	**0.03**
Anodal vs. Sham x Trial Type x Block 3 vs. (1, 2)	−0.25	0.78	−0.75	0.46
Anodal vs. Sham x Trial Type x Block 4 vs. (1, 2, 3)	0.06	1.06	0.17	0.86
Cathodal vs. Sham x Trial Type x Block 2 vs. 1	0.47	1.60	1.41	0.16
Cathodal vs. Sham x Trial Type x Block 3 vs. (1, 2)	−0.12	0.89	−0.37	0.71
Cathodal vs. Sham x Trial Type x Block 4 vs. (1, 2, 3)	0.50	1.65	1.46	0.14

*Boldfaced text indicates p < 0.05*.

a *OR: Odds Ratio*.

We also sought to ensure that the effect of anodal stimulation early in learning was not driven by the presence of DAC3 subjects. Controlling for DAC, the Anodal vs. Sham x Trial Type x Block 2 vs. 1 interaction remained significant (β = 0.86, *z* = 2.45, *p* = 0.01) and the Anodal vs. Sham x Block 2 vs. 1 interaction for CD remained at trend (β = −0.49, *z* = −1.74, *p* = 0.08), confirming that the effect was not driven by genotypic differences between stimulation conditions.

Taken together, these results indicate that anodal stimulation significantly impeded learning away from the instructions during the initial blocks. No other Anodal vs. Sham contrasts were significant (Table 2), including the overall effect of anodal stimulation (*p* = 0.97) and the Anodal vs. Sham x Trial Type interaction (*p* = 0.88), suggesting that anodal stimulation only weakly and transiently affected performance. In contrast to the anodal condition, there were no significant effects of cathodal stimulation (all *p*s > 0.14).

#### Uninstructed Learning

We also explored the effect of stimulation on accuracy during training for the uninstructed symbol pairs (AB, EF). Quantifying the effect of stimulation on uninstructed learning is important in order to show that effects on instruction are not in some way due to generally altered learning, especially given a prior report of altered performance on the AB pair under anodal stimulation (Turi et al., 2015).

Though there were no significant effects of stimulation condition at the *p* < 0.05 level, there was a trend-level Anodal vs. Sham x Trial Type interaction (β = 0.16, *z* = 1.66, *p* = 0.097; Figure 4A, Supplementary Table 13), reflecting somewhat better average performance on the AB pair by the anodal group. This difference is intriguing given increasing evidence that working memory processes contribute to RL performance (Collins and Frank, 2012; Collins et al., 2017), and anodal stimulation has been shown to improve working memory (Fregni et al., 2005; Zaehle et al., 2011; Nozari and Thompson-Schill, 2013). However, in light of the marginal nature of this unhypothesized effect, we do not interpret it further. As with instructed learning, there were no significant effects of cathodal stimulation (all *p*s > 0.12).

### tDCS: Test Phase

In contrast to the training phase, there were no significant effects of stimulation on either Avoid-D/Avoid-F or DF trials at test (all *p*s > 0.19; Figures 4C,D). This suggests that unlike COMT genotype, to the extent that tDCS modulated instructed learning, it biased choice during training without impacting the learned value of the instructed stimulus.

### Computational Modeling

While the behavioral analyses above confirm the existence of instructional bias, they are only weakly informative with respect to the underlying mechanisms. Two classes of models have been suggested to account for instructional bias on the PST: models in which instructions bias striatal reward learning (learning bias models), and those in which instructions affect choice rather than learning (choice bias models; Doll et al., 2009). Prior work has provided weak evidence for a choice bias operating during training, while test phase performance has been best explained by a learning bias mechanism (Doll et al., 2009, 2011). Two results from the present study bear on this question. First, the early-developing, persistent bias of the DAC3 group during training, coupled with their exclusive choice of D over F at test, would seem to be more consistent with a choice bias during both phases. However, these effects could also plausibly arise from a very strong learning bias, making this interpretation far from definitive. Second, the unaltered performance by the anodal group at test also appears more consistent with tDCS influencing a choice bias early in training, though caution is warranted in interpreting a null result.

We therefore fit computational models to subjects' data—one learning bias model and two choice bias models—each of which encapsulates a different hypothesis about the nature of instructional control (see section Methods: Computational Modeling). Briefly, the *learning bias model* (Doll et al., 2009) assumes instructional bias arises from an increase in learning rate for gains and a decrease in learning rate for losses when the instructed symbol D is selected. The *Bayesian hypothesis testing model* (Doll et al., 2009) assumes that subjects veridically learn the reward value of D in a Bayesian fashion, but must have a certain level of confidence that the value of D is below chance before they reliably stop choosing it. We additionally implemented a novel choice bias model, the *decision bias model*, which assumes a standard RL learner with a fixed bias added to the value of D during choice. Finally, we fit a standard RL model, which tests the null hypothesis of no bias.

Contrary to prior work, both the training and test phase were best explained by the decision bias model (Table 3). However, while AIC strongly supported this model at both training and test, the protected exceedance probabilities and estimated model frequencies did not provide strong evidence that this model was more frequent in the population for the training phase than the Bayesian hypothesis testing model. We therefore examined the correlation between each model's bias parameter and performance on CD trials across training, in order to ascertain whether one or the other model better accounted for behavior on instructed learning trials. The ϕ parameter of the Bayesian hypothesis testing model was significantly correlated with performance on CD trials (*r*_(101)_ = −0.23, *p* = 0.02). However, the correlation between the ρ parameter of the decision bias model and CD performance was much stronger (*r*_(101)_ = −0.66, *p* < 0.0001), and the difference between the correlations was significant (Steiger's *Z* = −3.82, *p* = 0.0001). In accordance with our tentative hypothesis based on the behavioral results, we conclude that both training and test phase performance can be parsimoniously accounted for by a single choice bias mechanism.

**Table 3:** Model comparison of reinforcement learning model fits to subject data.

**Model**	**FP**	**−LL**	**AIC**	**PEP**	**EF**
**TRAINING**					
Standard	3	13543.8	27705.7	0.007	0.239
Learning bias	5	13298.2	27626.4	5.081e-05	0.003
Bayes HT	4	13402.9	27629.8	0.339	0.362
Decision bias	4	13284.2	27392.4	0.654	0.397
**TEST**					
Standard	3	4755.4	10128.8	3.816e-16	0.164
Learning bias	5	4362.8	9755.7	3.816e-16	0.002
Bayes HT	4	4748.4	10320.8	3.816e-16	0.015
Decision bias	4	4304.6	9433.3	1.000	0.818

*FP: number of free parameters; −LL: negative log-likelihood; AIC: Akaike information criteria; PEP: protected exceedance probability, the probability that a given model is the most frequent in the population, above and beyond chance; EF: estimated model frequency, the frequency of the model in the population as estimated by the Bayesian random-effects analysis*.

We also reexamined genotypic and stimulation group differences with respect to the ρ parameter of the decision bias model. These results are reported fully in section 2 of the Supplementary Material and average parameter estimates are reported in Supplementary Tables 1 and 2. Briefly, we found effects of COMT and DAC on ρ at both training and test, in the same direction as the behavioral results. For DAT, 9-repeat carriers were fit with a higher ρ parameter during training, but test phase differences were best explained by the 9-repeat carrier group being fit with a lower learning rate for losses as compared to 10/10 group. We were, however, unable to confirm the anodal tDCS behavioral effect in the parameters of the decision bias model. While this does not invalidate the effect, it does warrant additional caution in interpreting the result.

## Discussion

There is mounting evidence that reward learning is far more complex and dynamic than can be accounted for by simple model-free theories of reinforcement. This complexity has been explored with respect to goal-directed planning processes (i.e., model-based RL; Dolan and Dayan, 2013) and instructional control (Wolfensteller and Ruge, 2012), among others. Both model-based RL and instructional control have been associated with cognitive control and frontostriatal function (Daw et al., 2005; Doll et al., 2009, 2011, 2014; Li et al., 2011; Wolfensteller and Ruge, 2012; Fouragnan et al., 2013; Smittenaar et al., 2013; Otto et al., 2015). While the importance of cognitive control to healthy cognitive functioning is indisputable, top-down control can be detrimental to learning and cognitive flexibility (Chrysikou et al., 2014; Gopnik et al., 2015).

In the case of instructed reinforcement learning, increased top-down control can be detrimental in that it leads to greater instructional bias toward inaccurate instructions. This study expands on the finding that instructional bias is associated with dopaminergic genes affecting PFC and striatal function (Doll et al., 2011), suggesting that the balance between PFC DA (COMT) and striatal DA (DAT1) modulates instructed learning. We further establish a causal link between PFC and biases found in instructed RL. In accord with our hypothesis, anodal subjects demonstrated more protracted learning away from the instructions during the early blocks of training, complementing the genetic evidence that individual differences associated with PFC function are linked to individual differences in instructional control of RL.

### A Dopamine Genetic Composite Is Associated With Instructed Learning

While both COMT Met/Met genotype and DAT1 9-repeat carrier genotype were individually significant predictors of greater instructional control during training, the DA composite revealed that this effect was selective to Met/Met:9-repeat carriers (DAC3). This greater bias emerged early in training and persisted throughout the training phase, unaffected by feedback. During test, a gene-dose effect, confirmed both within each gene and with the composite, demonstrated greater bias with increasing Met alleles and decreasing DAT1 repeats. These results are consistent with the known reciprocal relationship between PFC and striatal DA (Kolachana et al., 1995; King et al., 1997; Meyer-Lindenberg et al., 2005). It has been hypothesized that the balance between cognitive stability and cognitive flexibility is mediated via corticostriatal interactions and the differential modulation of prefrontal and striatal circuits by DA. While increases in prefrontal relative to striatal DA have been linked to cognitive stability, increases in striatal relative to prefrontal DA have been linked to cognitive flexibility (Cools and D'Esposito, 2009). We propose that increasing PFC DA, indexed by increasing Met alleles, coupled with decreasing tonic striatal DA, indexed by decreasing DAT1 repeats, shifts the balance away from bottom-up striatal learning based on reward prediction errors and toward PFC-mediated top-down control of RL.

While extracellular DA is primarily recycled via reuptake by DAT in striatal regions, there is little DAT expression in PFC, where levels of DA are controlled by reuptake via the norepinephrine transporter (NET) and enzymatic breakdown via COMT (Seamans and Yang, 2004; Sulzer et al., 2016). With regard to COMT, PFC DA plays a critical role in stabilizing working memory representations (Durstewitz and Seamans, 2008), which are thought to facilitate top-down control (Miller and Cohen, 2001). Notably, carriers of the Met allele of the Val158Met genetic polymorphism have diminished COMT enzyme activity and concomitantly higher levels of prefrontal dopamine (see Tunbridge, 2010 for review). Elevated DA in PFC may then cause increased D1 receptor stimulation, which further drives activity in PFC afferents such as the striatum (Bilder et al., 2004). Indeed, frontostriatal functional connectivity varies with COMT genotype (Tan et al., 2007; Krugel et al., 2009; Tunbridge et al., 2013). Behaviorally, the Met allele has been associated with enhanced working memory and cognitive control (see Witte and Flöel, 2012 for review). Carriers of the Val allele have more rapid breakdown of prefrontal dopamine and thus somewhat weaker working memory, but potentially greater cognitive flexibility (Krugel et al., 2009; Witte and Flöel, 2012). Replicating previous findings (Doll et al., 2011), the Met allele in our study was associated with greater instructional bias and therefore indicative of greater top-down control.

In the case of the DAT1/SLC6A3 VNTR, our behavioral results are consistent with increased DAT expression with the 9-repeat allele (Faraone et al., 2014) leading to reductions in tonic DA concentrations in the striatum. Reduced tonic DA in striatum has been shown to facilitate PFC input (Goto and Grace, 2005), which would in turn allow for greater biasing of RL. Furthermore, human imaging studies have demonstrated that DAT1 and COMT affect activity in prefrontal and striatal regions during reward anticipation. While the results of these studies are not entirely consistent, anticipatory activity in striatum is generally greater for DAT1 9-repeat carriers and is modulated by COMT genotype (Dreher et al., 2009; Aarts et al., 2010; cf. Yacubian et al., 2007), with one study finding the highest activity in both lateral PFC and ventral striatum for Met/Met:9-repeat carriers (Dreher et al., 2009).

However, this interpretation must be qualified by the considerable uncertainty surrounding the effect of the DAT1/SLC6A3 VNTR on dopaminergic function. Both *in vivo* and *in vitro* studies have produced conflicting results, with some supporting greater DAT expression for the 9-repeat allele compared to the 10-repeat allele, while others report the opposite, or no relationship (Costa et al., 2011; Faraone et al., 2014). A recent meta-analysis of human imaging studies supports the first possibility when restricting the analysis to normal controls (Faraone et al., 2014). Disease status, development, and ancestry may all play a role in the functional consequences of DAT1 (Franke et al., 2010; Shumay et al., 2011; Faraone et al., 2014). Even in the absence of changes in overall DAT expression, heterogeneities in DAT density and variations in neuronal morphology can substantially affect dopamine reuptake, which could contribute to the diversity of findings (Kaya et al., 2018).

It is also unclear the extent to which variation in DAT expression should be expected to influence tonic vs. phasic DA. Phasic DA bursts are associated with salient stimuli and have been shown to be associated with learning via reward prediction errors (Schultz et al., 1997; Berridge, 2012). Various roles have been ascribed to tonic DA, including modulation of response vigor (Niv et al., 2007), exploration (Beeler et al., 2010), and the relative weighting of effort costs (Salamone et al., 2007). DAT has a clear role in maintaining tonic DA concentrations (Efimova et al., 2016; Sulzer et al., 2016). Accordingly, DAT has been attributed a major role in synaptic DA clearance after phasic release (Bilder et al., 2004), and pharmacological blockade of DAT alters DA transients and leads to long lasting increases in tonic DA (Floresco et al., 2003; Ford et al., 2010). However, detailed biophysical modeling suggests that diffusion is responsible for synaptic clearance of DA, with DAT having a (potentially limited) role in shaping the radius and duration at which DA bursts could activate receptors via volume transmission (Cragg and Rice, 2004; Arbuthnott and Wickens, 2007; Rice and Cragg, 2008). Notably, increasing burst firing of DA neurons in the ventral tegmental area does not cause tonic increases in extracellular DA in the nucleus accumbens without DAT blockade (Floresco et al., 2003). Tonic DA may also indirectly influence phasic activity, though the direction of this influence is complicated to determine; elevated tonic DA due to increased tonic DA neuron firing may augment the peak and duration of DA bursts (Dreyer et al., 2010), but tonic concentrations may also inhibit phasic DA via autoreceptor feedback mechanisms (Bilder et al., 2004).

The performance of patients with schizophrenia provides an interesting counterpoint to the combined effect of COMT and DAT. Opposite to the Met/Met:9-repeat carrier genotype, the pathology of schizophrenia includes hyperdopaminergic tone in striatum and hypodopaminergic tone in PFC (Weinberger et al., 1992; da Silva Alves et al., 2008; Brisch et al., 2014; Slifstein et al., 2015; Grace and Gomes, 2018). Notably, patients with schizophrenia demonstrate reduced instructional bias on the PST (Doll et al., 2014). They also seem to rely less on putatively PFC-mediated processes in uninstructed learning, including reduced use of win-stay, lose-shift strategies and poorer performance on the easiest AB pair, potentially indicative of a reduced tendency to maximize or otherwise use rule-based strategies (Waltz et al., 2007, 2011; Doll et al., 2014). Though the elevated performance on AB in the Met/Met:9-repeat carrier group in the present study was not significant, it provides further evidence of opposite behavioral effects of opposite dopaminergic profiles.

Our findings of reduced flexibility with increasing ratio of PFC to striatal DA are also in accord with the effects of COMT and DAT1 on reversal learning. Compared to Met homozygotes, Val homozygotes show greater learning-rate adaptation around reversals, leading to improved performance (Krugel et al., 2009). Notably, Val homozygotes have more differentiated prediction error signals in striatal regions and greater learning-rate-dependent modulation of frontostriatal connectivity, suggestive of more adaptive prefrontal modulation of striatal RL (Krugel et al., 2009). On the other hand, the DAT1 9-repeat allele is associated with greater perseveration after a reversal (den Ouden et al., 2013). It is interesting to note that this perseveration effect was explained by the 9-repeat allele conferring a more rapidly decreasing learning rate with increasing experience, which may be related to the decreased learning rate modulation of COMT Met homozygotes. Direct comparison is difficult, however, as different computational models were used in the two studies. Importantly, while den Ouden and colleagues attributed their findings to more robust striatal learning of the previous reward contingencies, in the case of Met/Met:9-repeat carriers in the present study, their performance in the training phase cannot be due to greater ingraining of previous experience; the bias in the present case was due to instruction, not experience, was robustly evident in the first training block, and persisted throughout training.

### Stimulation Weakly Increased Instructional Bias

In contrast to the genetic effects, the effect of tDCS on performance was far more limited. In accord with our hypothesis, anodal subjects demonstrated modestly more protracted learning away from the instructions during the early blocks of training. However, there was no effect of cathodal stimulation, and no effect of either stimulation condition during the test phase.

While the isolation of the effect to the training phase makes sense in light of the postulated division between frontal and striatal systems during training and test (Frank et al., 2007), it is at odds with the finding of increased bias at test associated with the COMT Met allele. It may be the case that genotypic effects on frontostriatal DA balance or frontostriatal connectivity (discussed above) allow for greater biasing of striatum by PFC than is possible with single-session tDCS.

### Mechanisms of Instructional Bias

The mechanisms underlying instructional bias are under debate. Proposals include models in which instructions bias striatal reward learning (learning bias models; Biele et al., 2009; Doll et al., 2009) or those in which instructions affect choice rather than learning (choice bias models; Doll et al., 2009). Evidence in favor of each of these classes of models has been mixed. Past computational modeling has tended to support learning bias models (Biele et al., 2009, 2011; Doll et al., 2009, 2011) but does not unequivocally rule out choice bias models (Doll et al., 2009, 2011). A number of neuroimaging studies have favored neither class of models, finding blunted activation in basal ganglia structures during instructed/prior knowledge conditions, suggesting a suppression of RL (Delgado et al., 2005; Biele et al., 2011; Li et al., 2011; Fouragnan et al., 2013). However, one study found overall decreased activity in reward structures but activity consistent with a learning bias in the form of an “outcome bonus” for choosing the instructed stimulus (Biele et al., 2011).

Adding to this debate, we find that our training phase results can be explained by a novel choice bias model—the decision bias model—containing a fixed bias for choosing the instructed symbol. This is in contrast to past work, which has found that a standard RL model without instructional bias best fits training phase performance, despite clear behavioral effects of instruction during training (Doll et al., 2009, 2011). Our model also better predicted behavioral performance on CD trials compared to the Bayesian hypothesis testing model, a choice bias model previously shown to provide a reasonable fit to some subjects' training data and to be sensitive to effects of COMT (Doll et al., 2009, 2011). These results thus provide stronger evidence for the existence of a choice bias mechanism during training.

The decision bias and Bayesian hypothesis testing models differ in a number of regards (see section Methods: Computational Modeling), with the most prominent differences being in the type of learner (standard Q-learning vs. Bayesian Q-learning) and in the nature of the bias (fixed vs. variable). We cannot say with certainty which of these factors most contributes to the superior performance of the decision bias model, though comparing our pattern of results to past work suggests that the Bayesian learner detracted from the performance of the model; all else equal, a variable bias should presumably better capture the behavior of a putative fixed bias agent than no bias. That said, an important direction for future work is to introduce a variable bias into the standard Q-learning framework and compare this to a fixed bias. This poses a challenge, since the uncertainty information used to implement adaptivity in the Bayesian framework is not present in the standard framework.

Again contrary to prior results, the decision bias model also best explained performance at test. While model comparison and striatal dopaminergic genetic effects have been previously taken as evidence of a learning bias mechanism at test (Doll et al., 2009, 2011), the supposition that the test phase primarily measures learning free of choice effects has recently come into question (Shiner et al., 2012; Smittenaar et al., 2012), in keeping with a broader role of DA in modulating motivation and learned value representations (Cagniard et al., 2006; Berridge, 2012; Medic et al., 2014). Further supporting our finding, a recent reevaluation of test phase performance using an alternative model redescribed the learning bias for one striatal genotype as a choice bias (Collins and Frank, 2014). These discrepancies highlight the fact that model comparison results are dependent on the models tested. Additionally, in light of the evidence from other studies, there is no reason to think choice bias and learning bias mechanisms are mutually exclusive. However, the complexity of a model implementing both forms of bias would likely pose identifiability issues. We suggest that along with continued refinements to computational models, novel experimental designs capable of teasing apart these different possibilities will be necessary to advance our understanding of the mechanisms of instructional control.

### Specificity of the Effects and Limitations

While there is good evidence that the expression of COMT and DAT1 are regionally specific, caution must be taken in interpreting the results of stimulation, as the lack of focality of tDCS prevents strong claims about effects on specific brain regions. Stimulation could have altered the function of other brain areas involved in RL, including orbitofrontal cortex (O'Doherty, 2004). Neuroimaging and current modeling have even shown tDCS effects in subcortical structures, including the basal ganglia (Sadleir et al., 2010; Weber et al., 2014). However, the lack of stimulation effects on uninstructed learning and test phase performance somewhat militates against these possibilities.

Importantly, while our sample size was large for a tDCS study (Minarik et al., 2016) and was larger than the original report of the effects of COMT on instructed RL (Doll et al., 2011), these results should be replicated, particularly in light of the weakness of the tDCS effects and the small sample size of some genotypes. In the latter case, the low frequencies of the COMT Met and DAT1 9-repeat alleles in the population make collecting adequate samples of these groups challenging (Vandenbergh et al., 1992; Doucette-Stamm et al., 1995; Auton et al., 2015). Because access to such samples is difficult outside of large cohort studies, we took statistical steps within our sample to ensure the robustness of our genetic results. Given the known interaction of COMT and task on the effects of prefrontal stimulation (Plewnia et al., 2013; Nieratschker et al., 2015), larger samples would also permit an examination of genotype x stimulation interactions. Though a between-subjects design was necessary in this study due to the use of deception, future examinations of this topic could also be improved by the development of within-subjects designs. Finally, it is conceivable that there is more opportunity to decrease bias than increase it, given the overwhelming feedback subjects receive in contradiction to the instructions. Unfortunately, cathodal tDCS, which could in principle be used to test this hypothesis, failed to elicit an effect in the present case and is demonstrably unreliable (Jacobson et al., 2012; Nozari et al., 2014). Future studies using theta-burst transcranial magnetic stimulation may be an appropriate alternative.

### Conclusion

In sum, the present study provides further evidence for the role of PFC in biasing instructed RL, and additionally highlights the importance of frontostriatal DA balance in modulating top-down inputs. Such top-down regulation of learning by PFC is consistent with increased cognitive control leading to both costs and benefits (Chrysikou et al., 2014). Understanding the interplay of cognitive control and learning is thus key to establishing what level of control is most adaptive in a given situation. This endeavor will ultimately require delineating the relationship between computational and neurocognitive factors in learning and choice.

## Data Availability Statement

The raw data supporting the conclusions of this manuscript are available from the corresponding author upon reasonable request.

## Author Contributions

NT and ST-S formulated the experiment. NT implemented the experiment and performed data analysis. NT and KG collected the data. NT wrote the first draft of the manuscript, and all authors contributed to manuscript revision.

### Conflict of Interest Statement

The authors declare that the research was conducted in the absence of any commercial or financial relationships that could be construed as a potential conflict of interest.

## Footnotes

^1^In order to prevent learning rates exceeding 1.0, the learning bias parameters were also constrained such that $\alpha_{\text{b}\cdot }\leq \alpha_{.}^{-1}$ (Doll et al., 2009).
