# Supplementary material for: The Role of Frontostriatal Systems in Instructed Reinforcement Learning: Evidence From Genetic and Experimentally-Induced Variation
Source: Front Hum Neurosci. 2018 Dec 17;12:472. doi: 10.3389/fnhum.2018.00472 (PMC6304395; doi:10.3389/fnhum.2018.00472)
Supplement: Supplementary file 1 [file Data_Sheet_1.pdf]

# *Supplementary Material*

# The Role of Frontostriatal Systems in Instructed Reinforcement Learning: Evidence from Genetic and Experimentally-Induced Variation

**Nathan Tardiff*, Kathryn N. Graves, & Sharon L. Thompson-Schill**

*** Correspondence:** Nathan Tardiff: ntardiff@sas.upenn.edu

## 1 Supplementary Methods

### 1.1 Task Procedure

Subjects completed an instructed probabilistic selection task (iPST), presented on a 13” laptop computer via PsychoPy (Peirce, 2009). This task required subjects to learn the value of symbols initially presented in 3 pairs (AB, CD, EF; see Table 1, main text). Within each pair, one symbol had a higher probability of reward, and subjects were expected to learn to select the more highly rewarded symbols via feedback learning. Symbols were rendered as Japanese Hiragana characters and the assignment of Japanese character to underlying stimulus was randomized across subjects.

While seated in front of the computer, subjects first read the following instructions:

> Thank you for participating! Two black symbols will appear simultaneously on the computer screen. One symbol will be “correct” and the other will be “incorrect”, but at first you won’t know which is which. There is no ABSOLUTE right answer, but some symbols have a higher chance of being correct than others. Try to pick the symbol that you find to have the highest chance of being correct. You’ll have to figure out which symbols to select by testing them out. Note: the side of the screen on which a symbol appears does not affect its chances of being correct. Now you will be introduced to the symbols.

Each symbol was then presented individually for 5 sec each. When symbol D was presented the screen also displayed the following false advice: “This symbol has the best chance of being correct.”

Subjects were then tested on how many stimuli appear on every trial and how to choose the stimulus on the left or the right. Each symbol was then presented again for 5 sec and subjects were directed to press a key when the instructed symbol D was presented. The instructions restarted from the beginning until all questions were answered correctly.

During the training phase, subjects had to learn the value of each symbol via probabilistic feedback. On a given trial, subjects saw one of the three symbol pairs, side counterbalanced. Trials began with a fixation cross, followed by the stimulus display. Once a response was made, the selected symbol was highlighted via a square border, colored green for positive feedback and red for negative feedback. Additionally, symbolic feedback in the form of a green checkmark for positive feedback and a red cross-out mark for negative feedback was displayed centrally below the two symbols. Feedback was only provided for the selected option. In order to ensure consistency across subjects in the duration of the task relative to stimulation, all trials were fixed in length and proceeded as follows: 300 ms fixation, 2000 ms response window, 200 ms highlight time, 900 ms feedback time.

This was followed by a variable ITI (minimum 800 ms) calculated to bring the total duration of trial + ITI to 4200 ms. If subjects failed to make a response during the response window, a blue question mark was displayed in lieu of feedback for the remainder of the trial.

Subjects completed 4 training blocks. Each block contained 20 repetitions of each pair, for a total of 60 trials per block and 240 total training trials. Trial order and feedback were randomized within each block. Feedback was randomized such that within a block, each symbol was assigned reward at a rate equal to its underlying probability of reward (i.e., if the subject always chose symbol A, it would result in positive feedback on 16 of the 20 trials, for a p(reward) = 0.8). Feedback was also assigned in a complementary fashion within symbol pairs, such that in trials on which one symbol was assigned positive feedback the other symbol in the pair was assigned negative feedback.

After completing the training phase, the test phase began with the following instructions:

> It's time to test what you've learned! During this set of trials you will NOT receive feedback (correct or incorrect) on your responses. If you see new combinations of symbols in the test, please choose the symbol that "feels" more correct based on what you learned during the training sessions. If you're not sure which one to pick, just go with your gut instinct!

During the test phase, all possible symbol pairings were presented (e.g., AB, AC, AD, AE, AF, …) without feedback. Each pair was presented 6 times, for a total of 90 trials. Order was randomized across subjects.

### 1.2 Genotyping

DNA samples were collected via Oragene saliva kits (DNA Genotek) and extracted using the Chemagen MSMI DNA Extraction system. For the COMT Val158Met SNP, Taqman 5' nuclease PCR primers and probes were utilized (Life Technologies). Each probe consisted of an oligonucleotide with a fluorescent reporter dye, a non-fluorescent quencher and minor groove binder (MGB). Allele-specific cleavage of probes was detected using different reporter dyes for each probe (6FAM and VIC fluorophores for each allele), with separate wavelength maxima. PCR amplifications were set up in a 384-well plate format in total volume of 5 µl, containing 2.5 µl 2X universal master mix, 0.25 µl 1X primer and probe from ABI and 2.25 µl of DNA at a concentration of 5 ng/µl. Water as a negative control was included in each 384-well plate. PCR was performed in QuantStudio 12K Flex Real-Time PCR System (ABI). After an enzyme activation step for 10 min at 95 °C, 60 two-step cycles were performed; 15 sec denaturation at 95 °C followed by 1 min annealing/extension at 60 °C for all variants. After PCR, end-point fluorescence levels of 6FAM and VIC were measured automatically in each well using V1.2.2 manufacturer's custom software (ABI). Allelic discrimination results were then graphed on a scatter plot contrasting reporter dye florescence (i.e., Allele X vs. Allele Y).

For the DAT1/SLC6A3 VNTR, extracted DNA was amplified using DAT1 VNTR specific primers (Forward primer: 5'-6FAM-TGT-GGT-GTA-GGG-AAC-GGC-CTG-AG-3'; Reverse primer: 5'-CTT-CCT-GGA-GGT-CAC-GGC-TCA-AGG-3'; ABI #450007) utilizing the Roche Expand High Fidelity PCR System (#04738268001). Capillary electrophoresis was performed on the ABI 3130xl DNA Analyzer running POP7 polymer. One µl of amplified sample was suspended in 9 µl of Hi-Di Formamide (ABI #4311320) and 0.5 µl of Genescan-600 LIZ Size Standard v2.0 (ABI #4408399) and denatured at 95 °C for 2 min then placed on ice for an additional 2 min before loading onto the instrument. After electrophoresis, samples were analyzed using ABI Genemapper 4.0 software (Life Technologies).

## 2 Supplementary Results

Here we report the results of between-group parameter comparisons of the decision bias model (see sections Methods: Computational Modeling and Results: Computational Modeling of the main text for modeling details). These analyses complement the behavioral analyses in the main results of the paper, asking whether genotype and stimulation groups differ in the degree of their choice bias, as quantified by the $\rho$ parameter of the model. See Supplementary Tables 1 and 2 for average parameter estimates for each group.

### 2.1 COMT

Mirroring the training phase behavioral results, we found a significant effect of COMT status on $\rho$ ($F(2,99) = 3.31$, $p = .04$). The Met/Met group had a significantly larger bias than both Val/Val ($t(99) = 2.45$, $p_{corrected} = .049$) and Val/Met ($t(99) = 2.34$, $p_{corrected} = .049$). There was no difference between Val/Val and Val/Met ($t(99) = 0.34$, $p_{corrected} = .74$). We also found a significant gene-dose effect, whereby increasing Met alleles lead to increases in $\rho$ ($r(100) = .21$, $p = .04$).

Test phase fit results were similar to those on the Avoid-D/Avoid-F measure. There was not a significant effect of COMT on $\rho$ ($F(2,99) = 2.10$, $p = .13$). There was, however, a significant gene-dose effect ($r(100) = .20$, $p = .04$), whereby increasing Met alleles were associated with increasing bias.

### 2.2 DAT

Though 9-repeat carriers were on average fit with a higher value of $\rho$ than 10/10 homozygotes ($M_{9c} = 0.20$, $M_{10/10} = 0.12$), this difference was only significant at a trend level ($t(100) = 1.90$, $p = .06$). Additionally, no other model parameters better explained the difference in instructed training phase performance (all $p$s > .45).

The inability to find a significant difference in model parameters despite a significant difference in behavioral performance could indicate that the decision bias model merely fails to capture the relevant difference between DAT groups. It may be the case, however, that noise in the parameter estimates masks a significant group difference. One common source of noise in such estimates is correlations among the parameters. We therefore asked whether 9-repeat carriers would have a significantly greater value of $\rho$, controlling for the other model parameters. Indeed they did ($\beta = 0.10$, $t(97) = 2.68$, $p = .009$)[1].

We did not find that DAT modulated the $\rho$ parameter at test ($t(100) = 1.03$, $p = .31$). However, examining the other parameters, we found that 9-repeat carriers had a significantly lower learning rate for losses ($\alpha_l$) than 10/10 homozygotes ($t(100) = -2.75$, $p = .007$). No other differences were significant (all $p$s > .14). This difference in $\alpha_l$ is in keeping with the main effect of DAT on Avoid-D/Avoid-F performance in the absence of a significant interaction, though only the difference in Avoid-D was individually significant.

One question raised here is why a lower learning rate for losses would produce worse performance, when past investigations have demonstrated that a lower learning rate for losses can produce better performance in avoidance learning due to more stably learned values (Frank et al., 2007). One possibility is that the 9-repeat carriers were to a greater extent fit by *very* low $\alpha_l$, such that learning was impaired. Indeed, a greater proportion of

---

[1] In light of this finding, we repeated all other group comparisons of the $\rho$ parameter, controlling for the other parameters. The significance of all other comparisons were largely unchanged, excepting following: the COMT gene-dose effect at test fell to a trend level ($p = .055$), and the effect of DAC at test rose to a trend level ($p = .052$).

9-repeat carriers were fit with $\alpha_l < .01$ compared to 10/10 homozygotes (9c: 45.7%, 10/10: 25.4%; $p = .046$, Fisher's Exact Test).

If low $\alpha_l$ impaired learning, this should be reflected in the Q-values produced by the model. This was the case. In addition to a main effect of Symbol, indicating that overall Q-values were differentiated among the symbols ($F(5,500) = 95.49$, $p < .0001$), we also found a main effect of DAT ($F(1,100) = 9.41$, $p = .003$), qualified by a Symbol x DAT interaction ($F(5,500) = 2.83$, $p = .016$). Post-hoc tests revealed that all symbol values were inflated in the 9-carrier group relative to 10/10, and all these differences were significant except that for symbol A ($A_{9c\text{-}10/10}$: M = 0.06, $t(171.82) = 1.06$, $p_{corrected} = .29$; $B_{9c\text{-}10/10}$: M = 0.17, $t(171.82) = 2.89$, $p_{corrected} = .017$; $C_{9c\text{-}10/10}$: M = 0.14, $t(171.82) = 2.36$, $p_{corrected} = .04$; $D_{9c\text{-}10/10}$: M = 0.19, $t(171.82) = 3.34$, $p_{corrected} = .005$; $E_{9c\text{-}10/10}$: M = 0.16, $t(171.82) = 2.69$, $p_{corrected} = .02$; $F_{9c\text{-}10/10}$: M = 0.21, $t(171.82) = 3.65$, $p_{corrected} = .002$).

These results suggest that the distortion of Q-values in the 9-carrier group affected negatively valued stimuli (B, D, F) greater than positively valued stimuli (A, C, E). This in turn could have affected the overall spread in value between negative and positive stimuli. Indeed, while both groups valued positive stimuli more than negative stimuli (9c: $M_{pos\text{-}neg} = 0.21$, $t(100) = 7.03$, $p_{corrected} < .0001$; 10/10: $M_{pos\text{-}neg} = 0.28$, $t(100) = 13.12$, $p_{corrected} < .0001$), this difference was reduced in 9-repeat carriers ($F(1,100) = 3.96$, $p = .049$). This still does not explain, however, why 9-carriers were (nonsignificantly) more impaired on Avoid-D than Avoid-F. Though there was no effect of DAT on $\rho$ at test, 9-repeat carriers were on average fit with a higher value (Supplementary Table 2). It may be the case that this small parameter difference was enough to produce a behavioral effect in the absence of a significant difference in the parameter.

These results are also illuminating with respect to the influence of DAT on phasic and tonic DA (see section A Dopamine Genetic Composite Is Associated With Instructed Learning of the main text). Other striatal genes assayed in this paradigm asymmetrically affect approach and avoidance learning, as measured by genotypic differences in learning rates for gains and losses, respectively (Doll et al., 2011; Frank et al., 2007). Such differences are taken to reflect differences in the efficacy of phasic DA to affect learning, as learning rates govern the extent to which reward prediction errors conveyed by phasic DA update learned stimulus values. In the training phase, the finding that 9-carriers were best characterized as having an increased choice bias relative to 10/10 homozygotes is consistent with our hypothesis of an effect of DAT1 on tonic DA. In the test phase, however, the decreased learning rate for losses for 9-repeat carriers—in the absence of a significant difference in decision bias—is better explained by an effect on phasic DA. One potential way to reconcile these differences between training and test would be if lower tonic DA in 9-repeat carriers produced less contrast in DA for the phasic dips thought to convey negative reward prediction errors (Niv et al., 2005).

## 2.3 DA Composite

There was a significant effect of DAC on $\rho$ at training ($F(3,98) = 4.10$, $p = .009$), in line with the behavioral results. Post-hoc comparisons revealed that the DAC3 group had a significantly higher bias than all other groups (DAC3 vs. DAC0: $t(98) = 3.50$, $p_{corrected} = .004$; DAC3 vs. DAC1: $t(98) = 3.00$, $p_{corrected} = .017$; DAC3 vs. DAC2: $t(98) = 2.86$, $p_{corrected} = .02$). No other comparisons reached significance (all $p_{corrected} > .84$). There was also a significant gene-dose effect, with increasing DAC status associated with increasing bias ($r(100) = .26$, $p = .009$).

Again mirroring the Avoid-D/Avoid-F results, the effect of DAC on $\rho$ during the test phase was not significant ($F(3,98) = 2.05$, $p = .11$). However, there was a significant gene-dose affect ($r(100) = .21$, $p = .03$).

## 2.4 tDCS

Though we found a significant effect of anodal stimulation during the training phase, there was no difference between the anodal and sham groups in the $\rho$ parameter of the model ($t(65) = 0.54$, $p = .59$). Nor was there a difference between cathodal and sham ($t(68) = 0.57$, $p = .57$). Nor did we find differences in any other model parameters (all *p*s > .36). Because the anodal effect was only present early during training, we also refit the decision bias model on just the first two blocks of training phase data. We again found no difference in $\rho$ between anodal and sham ($t(65) = -0.49$, $p = .63$). In keeping with the behavioral results, we also found no difference in $\rho$ during the test phase (Anodal vs. Sham: $t(65) = -0.56$, $p = .57$; Cathodal vs. Sham: $t(68) = 0.37$, $p = .71$).

In sum, we failed to find an effect of stimulation on model parameters. It may be that noise in the model parameter estimates prevented us from corroborating what was a very small behavioral effect at training for the comparison of anodal and sham. It may also be that anodal stimulation did not have a focal effect on any one parameter but rather induced weak, diffuse effects that together lead to a small behavioral difference in the absence of significant differences in model parameters (i.e., the numerically smaller learning rates and temperature parameters of the anodal group, combined with a numerically higher bias, could potentially have produced a small behavioral difference).

# 3 Supplementary Tables

## 3.1 Model Parameter Estimates

Supplementary Table 1. Parameter estimates for the decision bias model at training.

| Group | N | $\alpha_g$ | $\alpha_l$ | $\beta$ | $\rho$ |
|---|---|---|---|---|---|
| Overall | 103 | 0.34 (0.30) | 0.21 (0.27) | 0.29 (0.16) | 0.16 (0.20) |
| COMT | | | | | |
| Val/Val | 34 | 0.31 (0.30) | 0.22 (0.30) | 0.27 (0.17) | 0.12 (0.18) |
| Val/Met | 53 | 0.39 (0.31) | 0.20 (0.26) | 0.31 (0.17) | 0.14 (0.20) |
| Met/Met | 15 | 0.29 (0.28) | 0.18 (0.24) | 0.26 (0.15) | 0.27 (0.19) |
| DAT | | | | | |
| 10/10 | 67 | 0.34 (0.30) | 0.20 (0.26) | 0.30 (0.17) | 0.12 (0.15) |
| 9c | 35 | 0.36 (0.31) | 0.22 (0.29) | 0.27 (0.16) | 0.20 (0.25) |
| DA composite | | | | | |
| 0 | 25 | 0.31 (0.29) | 0.18 (0.26) | 0.27 (0.18) | 0.10 (0.13) |
| 1 | 43 | 0.35 (0.30) | 0.23 (0.29) | 0.30 (0.16) | 0.15 (0.20) |
| 2 | 27 | 0.42 (0.32) | 0.21 (0.28) | 0.31 (0.17) | 0.15 (0.20) |
| 3 | 7 | 0.19 (0.12) | 0.12 (0.17) | 0.20 (0.11) | 0.38 (0.22) |
| tDCS | | | | | |
| Anodal | 33 | 0.29 (0.28) | 0.20 (0.25) | 0.26 (0.15) | 0.17 (0.23) |
| Sham | 34 | 0.35 (0.31) | 0.21 (0.30) | 0.29 (0.17) | 0.14 (0.19) |
| Cathodal | 36 | 0.39 (0.31) | 0.21 (0.26) | 0.30 (0.17) | 0.16 (0.18) |

*Note.* Parameter estimates are given as M (SD). Genotype counts only add up to 102 because genotyping failed for one subject.

Supplementary Table 2. Parameter estimates for the decision bias model at test.

| Group | N | $\alpha_g$ | $\alpha_l$ | $\beta$ | $\rho$ |
|---|---|---|---|---|---|
| Overall | 103 | 0.28 (0.35) | 0.23 (0.33) | 0.20 (0.08) | 0.27 (0.31) |
| COMT | | | | | |
| Val/Val | 34 | 0.24 (0.33) | 0.18 (0.31) | 0.19 (0.09) | 0.19 (0.25) |
| Val/Met | 53 | 0.32 (0.35) | 0.28 (0.37) | 0.20 (0.07) | 0.29 (0.33) |
| Met/Met | 15 | 0.26 (0.39) | 0.19 (0.27) | 0.19 (0.08) | 0.37 (0.33) |
| DAT | | | | | |
| 10/10 | 67 | 0.28 (0.33) | 0.30 (0.36) | 0.21 (0.08) | 0.25 (0.30) |
| 9c | 35 | 0.29 (0.37) | 0.11 (0.23) | 0.18 (0.07) | 0.32 (0.32) |
| DA composite | | | | | |
| 0 | 25 | 0.26 (0.34) | 0.20 (0.30) | 0.19 (0.10) | 0.19 (0.26) |
| 1 | 43 | 0.26 (0.31) | 0.33 (0.39) | 0.20 (0.08) | 0.27 (0.31) |
| 2 | 27 | 0.36 (0.40) | 0.15 (0.27) | 0.22 (0.07) | 0.28 (0.32) |
| 3 | 7 | 0.20 (0.36) | 0.10 (0.08) | 0.13 (0.04) | 0.51 (0.35) |
| tDCS | | | | | |
| Anodal | 33 | 0.31 (0.36) | 0.31 (0.39) | 0.18 (0.07) | 0.23 (0.29) |
| Sham | 34 | 0.30 (0.33) | 0.21 (0.30) | 0.21 (0.09) | 0.27 (0.29) |
| Cathodal | 36 | 0.25 (0.35) | 0.19 (0.31) | 0.21 (0.08) | 0.30 (0.34) |

*Note.* Parameter estimates are given as M (SD). Genotype counts only add up to 102 because genotyping failed for one subject.

## 3.2 Demographics

Supplementary Table 3. Demographic breakdown of the 103 subjects included in the analyses after performance cutoffs.

| Race/Ethnicity | N |
|---|---|
| Caucasian | 50 |
| Asian | 24 |
| African American | 20 |
| Other | 9 |
| Hispanic | |
| Y | 14 |
| N | 87 |
| Unknown | 2 |

Supplementary Table 4. Demographic breakdown by tDCS condition.

| | Anodal | Cathodal | Sham |
|---|---|---|---|
| Age | | | |
| Mean | 21.82 | 21.92 | 21.76 |
| SD | 5.12 | 3.55 | 4.27 |
| Gender | | | |
| M | 10 | 17 | 11 |
| F | 23 | 19 | 23 |
| Race | | | |
| Caucasian | 15 | 23 | 12 |
| African American | 8 | 4 | 8 |
| Asian | 8 | 5 | 11 |
| Other | 2 | 4 | 3 |
| Ethnicity | | | |
| Hispanic | 4 | 5 | 5 |
| Non-Hispanic | 28 | 30 | 29 |
| Genotype | | | |
| Val/Met | 19 | 17 | 17 |
| Met/Met | 5 | 8 | 2 |
| Val/Val | 9 | 11 | 14 |
| DAT 9c | 12 | 14 | 9 |
| DAT 10/10 | 21 | 22 | 24 |

*Note.* Genotype counts only add up to 102 because genotyping failed for one subject.

Supplementary Table 5. Demographic breakdown by genotype.

| | COMT | | | DAT | |
|---|---|---|---|---|---|
| | Val/Met | Met/Met | Val/Vat | 9c | 10/10 |
| Age | | | | | |
| Mean | 22.70 | 21.80 | 20.62 | 21.09 | 22.28 |
| SD | 5.21 | 3.10 | 2.67 | 3.53 | 4.63 |
| Gender | | | | | |
| M | 24 | 6 | 7 | 14 | 23 |
| F | 29 | 9 | 27 | 21 | 44 |
| Race | | | | | |
| Caucasian | 26 | 13 | 10 | 21 | 28 |
| African American | 9 | 2 | 9 | 8 | 12 |
| Asian | 11 | 0 | 13 | 3 | 21 |
| Other | 7 | 0 | 2 | 3 | 6 |
| Ethnicity | | | | | |
| Hispanic | 7 | 1 | 6 | 7 | 7 |
| Non-Hispanic | 44 | 14 | 28 | 28 | 58 |
| tDCS Condition | | | | | |
| Anodal | 19 | 5 | 9 | 12 | 21 |
| Cathodal | 17 | 8 | 11 | 14 | 22 |
| Sham | 17 | 2 | 14 | 9 | 24 |

*Note.* Genotype counts only add up to 102 because genotyping failed for one subject.

Supplementary Table 6. Demographic breakdown by DA composite.

| | DAC0 | DAC1 | DAC2 | DAC3 |
|---|---|---|---|---|
| Age | | | | |
| Mean | 21.20 | 22.21 | 22.15 | 21.14 |
| SD | 2.84 | 5.33 | 4.09 | 2.04 |
| Gender | | | | |
| M | 4 | 17 | 15 | 1 |
| F | 21 | 26 | 12 | 6 |
| Race | | | | |
| Caucasian | 6 | 19 | 18 | 6 |
| African American | 6 | 8 | 5 | 1 |
| Asian | 12 | 10 | 2 | 0 |
| Other | 1 | 6 | 2 | 0 |
| Ethnicity | | | | |
| Hispanic | 5 | 3 | 5 | 1 |
| Non-Hispanic | 20 | 38 | 22 | 6 |
| tDCS Condition | | | | |
| Anodal | 5 | 18 | 7 | 3 |
| Cathodal | 8 | 11 | 15 | 2 |
| Sham | 12 | 14 | 5 | 2 |

*Note.* Genotype counts only add up to 102 because genotyping failed for one subject.

## 3.3 Training Phase Results

Supplementary Table 7. ANOVA table for the mixed effects logistic regression model of the effect of COMT on instructed (CD vs. EF) training phase performance.

| Predictor | $\chi^2$ | *df* | *p* |
|---|---|---|---|
| **Intercept** | **6.24** | **1** | **.01** |
| COMT | 4.11 | 2 | .13 |
| **Trial Type** | **66.24** | **1** | **< .0001** |
| **Block** | **14.78** | **3** | **.002** |
| **COMT x Trial Type** | **13.94** | **2** | **.0009** |
| COMT x Block | 2.74 | 6 | .84 |
| Trial Type x Block | 0.68 | 3 | .88 |
| COMT x Trial Type x Block | 7.83 | 6 | .25 |

*Note.* Boldfaced text indicates $p < .05$.

Supplementary Table 8. ANOVA table for the mixed effects logistic regression model of the effect of COMT on uninstructed (AB, EF) training phase performance.

| Predictor | $\chi^2$ | *df* | *p* |
|---|---|---|---|
| **Intercept** | **194.47** | **1** | **< .0001** |
| COMT | 2.75 | 2 | .25 |
| **Trial Type** | **76.99** | **1** | **< .0001** |
| **Block** | **34.39** | **3** | **< .0001** |
| COMT x Trial Type | 0.42 | 2 | .81 |
| COMT x Block | 6.91 | 6 | .33 |
| **Trial Type x Block** | **17.11** | **3** | **.0007** |

*Note.* Boldfaced text indicates $p < .05$.

Supplementary Table 9. Mixed effects logistic regression model of the effect of DAT on instructed (CD vs. EF) training phase performance.

| Predictor | *β* | *OR*[a] | *z* | *p* |
|---|---|---|---|---|
| **Intercept** | **-0.32** | **0.73** | **-3.28** | **.001** |
| **9c vs. 10/10** | **-0.43** | **0.65** | **-2.17** | **.03** |
| **Trial Type** | **1.00** | **2.72** | **7.18** | **< .0001** |
| **Block 2 vs. 1** | **0.30** | **1.35** | **2.47** | **.01** |
| **Block 3 vs. (1,2)** | **0.25** | **1.28** | **2.33** | **.02** |
| **Block 4 vs. (1,2,3)** | **0.30** | **1.35** | **2.81** | **.005** |
| 9c vs. 10/10 x Trial Type | 0.53 | 1.70 | 1.91 | .06 |
| 9c vs. 10/10 x Block 2 vs. 1 | -0.10 | 0.90 | -0.41 | .68 |
| 9c vs. 10/10 x Block 3 vs. (1,2) | 0.12 | 1.13 | 0.56 | .57 |
| 9c vs. 10/10 x Block 4 vs. (1,2,3) | 0.02 | 1.02 | 0.08 | .94 |
| Trial Type x Block 2 vs. 1 | -0.26 | 0.77 | -1.72 | .09 |
| Trial Type x Block 3 vs. (1,2) | -0.02 | 0.98 | -0.14 | .89 |
| Trial Type x Block 4 vs. (1,2,3) | -0.12 | 0.89 | -0.77 | .44 |
| 9c vs. 10/10 x Trial Type x Block 2 vs. 1 | -0.24 | 0.79 | -0.81 | .42 |
| 9c vs. 10/10 x Trial Type x Block 3 vs. (1,2) | 0.01 | 1.01 | 0.02 | .98 |
| 9c vs. 10/10 x Trial Type x Block 4 vs. (1,2,3) | 0.10 | 1.11 | 0.34 | .73 |

*Note.* Boldfaced text indicates $p < .05$. [a]*OR*: Odds Ratio

Supplementary Table 10. Mixed effects logistic regression model of the effect of DAT on uninstructed (AB, EF) training phase performance.

| Predictor | *β* | *OR*[a] | *z* | *p* |
|---|---|---|---|---|
| **Intercept** | **1.12** | **3.06** | **13.93** | **< .0001** |
| 9c vs. 10/10 | -0.004 | 1.00 | -0.03 | .97 |
| **Trial Type** | **0.46** | **1.58** | **9.8** | **< .0001** |
| **Block 2 vs. 1** | **0.40** | **1.49** | **4.38** | **< .0001** |
| **Block 3 vs. (1,2)** | **0.23** | **1.26** | **2.77** | **.006** |
| **Block 4 vs. (1,2,3)** | **0.19** | **1.21** | **2.09** | **.04** |
| 9c vs. 10/10 x Trial Type | 0.01 | 1.01 | 0.12 | .91 |
| 9c vs. 10/10 x Block 2 vs. 1 | -0.12 | 0.89 | -0.73 | .47 |
| 9c vs. 10/10 x Block 3 vs. (1,2) | 0.11 | 1.12 | 0.65 | .51 |
| 9c vs. 10/10 x Block 4 vs. (1,2,3) | 0.03 | 1.03 | 0.16 | .87 |
| **Trial Type x Block 2 vs. 1** | **0.33** | **1.39** | **4.10** | **< .0001** |
| Trial Type x Block 3 vs. (1,2) | 0.004 | 1.00 | 0.08 | .94 |
| Trial Type x Block 4 vs. (1,2,3) | 0.02 | 1.02 | 0.27 | .79 |

*Note.* Boldfaced text indicates $p < .05$. [a]*OR*: Odds Ratio

Supplementary Table 11. ANOVA table for the mixed effects logistic regression model of the effect of DAC on instructed (CD vs. EF) training phase performance.

| Predictor | $\chi^2$ | *df* | *p* |
|---|---|---|---|
| Intercept | 1.45 | 1 | .23 |
| **DAC** | **11.03** | **3** | **.01** |
| **Trial Type** | **85.33** | **1** | **< .0001** |
| **Block** | **8.68** | **3** | **.03** |
| **DAC x Trial Type** | **29.61** | **3** | **< .0001** |
| DAC x Block | 9.68 | 9 | .38 |
| Trial Type x Block | 2.10 | 3 | .55 |
| DAC x Trial Type x Block | 9.12 | 9 | .43 |

*Note.* Boldfaced text indicates $p < .05$.

Supplementary Table 12. ANOVA table for the mixed effects logistic regression model of the effect of DAC on uninstructed (AB, EF) training phase performance.

| Predictor | $\chi^2$ | *df* | *p* |
|---|---|---|---|
| **Intercept** | **174.86** | **1** | **< .0001** |
| DAC | 3.40 | 3 | .33 |
| **Trial Type** | **67.10** | **1** | **< .0001** |
| **Block** | **26.87** | **3** | **< .0001** |
| DAC x Trial Type | 0.69 | 3 | .88 |
| DAC x Block | 5.55 | 9 | .78 |
| **Trial Type x Block** | **16.99** | **3** | **.0007** |

*Note.* Boldfaced text indicates $p < .05$.

Supplementary Table 13. Mixed effects logistic regression model of the effect of tDCS on uninstructed (AB, EF) training phase performance.

| Predictor | *β* | *OR*[a] | *z* | *p* |
|---|---|---|---|---|
| **Intercept** | **1.12** | **3.06** | **14.67** | **< .0001** |
| Anodal vs. Sham | 0.09 | 1.09 | 0.55 | .58 |
| Cathodal vs. Sham | -0.15 | 0.86 | -0.88 | .38 |
| **Trial Type** | **0.46** | **1.58** | **10.55** | **< .0001** |
| **Block 2 vs. 1** | **0.40** | **1.49** | **4.79** | **< .0001** |
| **Block 3 vs. (1,2)** | **0.22** | **1.25** | **2.74** | **.006** |
| **Block 4 vs. (1,2,3)** | **0.18** | **1.20** | **2.17** | **.03** |
| Anodal vs. Sham x Trial Type | 0.16 | 1.17 | 1.66 | .10 |
| Cathodal vs. Sham x Trial Type | -0.05 | 0.95 | -0.48 | .63 |
| Anodal vs. Sham x Block 2 vs. 1 | 0.31 | 1.36 | 1.63 | .10 |
| Cathodal vs. Sham x Block 2 vs. 1 | 0.29 | 1.34 | 1.54 | .12 |
| Anodal vs. Sham x Block 3 vs. (1,2) | -0.06 | 0.94 | -0.34 | .74 |
| Cathodal vs. Sham x Block 3 vs. (1,2) | -0.04 | 0.96 | -0.19 | .85 |
| Anodal vs. Sham x Block 4 vs. (1,2,3) | 0.07 | 1.07 | 0.38 | .70 |
| Cathodal vs. Sham x Block 4 vs. (1,2,3) | 0.10 | 1.11 | 0.52 | .60 |
| **Trial Type x Block 2 vs. 1** | **0.31** | **1.36** | **3.97** | **< .0001** |
| Trial Type x Block 3 vs. (1,2) | 0.02 | 1.02 | 0.26 | .80 |
| Trial Type x Block 4 vs. (1,2,3) | 0.02 | 1.02 | 0.33 | .74 |

*Note.* Boldfaced text indicates $p < .05$. [a]*OR*: Odds Ratio

### 3.4 References

Doll, B. B., Hutchison, K. E., and Frank, M. J. (2011). Dopaminergic genes predict individual differences in susceptibility to confirmation bias. *J. Neurosci.* 31, 6188–6198. doi:10.1523/JNEUROSCI.6486-10.2011.

Frank, M. J., Moustafa, A. A., Haughey, H. M., Curran, T., and Hutchison, K. E. (2007). Genetic triple dissociation reveals multiple roles for dopamine in reinforcement learning. *Proc. Natl. Acad. Sci.* 104, 16311–16316. doi:10.1073/pnas.0706111104.

Niv, Y., Duff, M. O., and Dayan, P. D. (2005). Dopamine, uncertainty and TD learning. *Behav. Brain Funct.* 1, 6. doi:10.1186/1744-9081-1-6.

Peirce, J. W. (2009). Generating stimuli for neuroscience using PsychoPy. *Front. Neuroinform.* 2, 10. doi:10.3389/neuro.11.010.2008.
